# Sex differences in paternal arsenic-induced intergenerational metabolic effects are mediated by estrogen

**DOI:** 10.1186/s13578-023-01121-4

**Published:** 2023-09-10

**Authors:** Yanfeng Xue, Yingyun Gong, Xin Li, Fei Peng, Guolian Ding, Zhao Zhang, Junchao Shi, Ilma Saleh Savul, Yong Xu, Qi Chen, Leng Han, Shengyong Mao, Zheng Sun

**Affiliations:** 1 College of Animal Science and Technology, Anhui Agricultural University, Hefei, China; 2 National Center for International Research on Animal Gut Nutrition, Center for Ruminant Nutrition and Feed Technology Research, College of Animal Science and Technology, Nanjing Agricultural University, Nanjing, China; 3 Division of Endocrinology, Department of Medicine, Baylor College of Medicine, Houston, TX USA; 4 Department of Endocrinology and Metabolism, the First Affiliated Hospital of Nanjing Medical University, Nanjing, China; 5 Obstetrics and Gynecology Hospital, Institute of Reproduction and Development, Fudan University, Shanghai, China; 6 Department of Biochemistry and Molecular Biology, McGovern Medical School, University of Texas Health Science Center at Houston, Houston, TX USA; 7 Molecular Medicine Program, Department of Human Genetics, and Division of Urology, Department of Surgery, University of Utah School of Medicine, Salt Lake City, UT USA; 8 Children’s Nutrition Research Center, Department of Pediatrics, Baylor College of Medicine, Houston, TX USA; 9 Department of Molecular and Cellular Biology, Baylor College of Medicine, Houston, TX USA

**Keywords:** Arsenic exposure, Environmental health, Glucose and lipid metabolism, Estrogen signaling pathway, Epigenetic inheritance

## Abstract

**Background:**

Gene-environment interactions contribute to metabolic disorders such as diabetes and dyslipidemia. In addition to affecting metabolic homeostasis directly, drugs and environmental chemicals can cause persistent alterations in metabolic portfolios across generations in a sex-specific manner. Here, we use inorganic arsenic (iAs) as a prototype drug and chemical to dissect such sex differences.

**Methods:**

After weaning, C57BL/6 WT male mice were treated with 250 ppb iAs in drinking water (iAsF0) or normal water (conF0) for 6 weeks and then bred with 15-week-old, non-exposed females for 3 days in cages with only normal water (without iAs), to generate iAsF1 or conF1 mice, respectively. F0 females and all F1 mice drank normal water without iAs all the time.

**Results:**

We find that exposure of male mice to 250 ppb iAs leads to glucose intolerance and insulin resistance in F1 female offspring (iAsF1-F), with almost no change in blood lipid profiles. In contrast, F1 males (iAsF1-M) show lower liver and blood triglyceride levels than non-exposed control, with improved glucose tolerance and insulin sensitivity. The liver of F1 offspring shows sex-specific transcriptomic changes, with hepatocyte-autonomous alternations of metabolic fluxes in line with the sex-specific phenotypes. The iAsF1-F mice show altered levels of circulating estrogen and follicle-stimulating hormone. Ovariectomy or liver-specific knockout of estrogen receptor α/β made F1 females resemble F1 males in their metabolic responses to paternal iAs exposure.

**Conclusions:**

These results demonstrate that disrupted reproductive hormone secretion in alliance with hepatic estrogen signaling accounts for the sex-specific intergenerational effects of paternal iAs exposure, which shed light on the sex disparities in long-term gene-environment interactions.

**Supplementary Information:**

The online version contains supplementary material available at 10.1186/s13578-023-01121-4.

## Highlights

Paternal iAs causes sex-specific effects on glucose and lipid metabolism in F1 offspring.

Paternal iAs causes sex-specific transcriptomic changes in the liver of F1 offspring.

Hepatocytes from the F1 offspring show sex-specific, cell-autonomous alterations in metabolic fluxes.

Paternal iAs disrupts reproductive hormone levels in F1 females.

Gonadal hormones are required for the sex-specific effects of paternal iAs.

Estrogen receptor in the F1 liver is required for the manifestation of the sex difference.

**Supplementary Information:**

The online version contains supplementary material available at 10.1186/s13578-023-01121-4.

## Background

Gene-environment interactions play essential roles in metabolic disorders such as diabetes and dyslipidemia. In addition to dietary and sedentary lifestyles, industrial or pharmaceutical chemicals are increasingly recognized as a contributor to the pandemic of metabolic disorders [1, 2]. Intriguingly, diet and chemicals not only affect metabolic homeostasis directly but also can cause persistent alterations in metabolic portfolios across generations [3]. The intergenerational effects of exposure to pharmaceutical or industrial chemicals are particularly relevant to human health. On one hand, the potential adverse intergenerational effects of drugs are not routinely tested, making it a blind spot for drug toxicity. On the other hand, industrialization has caused widespread pollution of soil, water, air, and food, which is associated with an elevated incidence of metabolic disorders [4, 5]. Although pollution has been declining in developed countries in recent decades [6, 7], its long-lasting effects might persist across generations and still contribute to chronic diseases in the current population. The maternal effects during prenatal or *in-utero* environmental exposure have been known as the Developmental Origins of Health and Disease (DOHaD) paradigm [8], while the paternal effects have begun to gain recognization recently [9].

Inorganic arsenic (iAs) is a medicine and a common environmental chemical. It is on the World Health Organization’s List of Essential Medicines [10] and the top hazardous substance in the Agency for Toxic Substances and Disease Registry. About 50,000 tonnes of arsenic trioxide are produced a year [11]. As a drug, iAs has a long history of being a traditional medicine in Asian countries [12] and is currently used to treat leukemia around the globe. As an industrial chemical, iAs was used in forestry products, glass production, wood preservative, and electronics [11]. As an environmental toxin, iAs is widely found in soil and drinking water. iAs can cause metabolic disorders upon direct exposure [13]. The maternal effects of iAs have been characterized under the DOHaD paradigm [14–24]. These maternal iAs effects might be attributable to disrupted embryo development or altered maternal nurturing behaviors [25]. We have recently reported the intergenerational effects of iAs exposure in mice in an exclusive paternal exposure diagram [26]. We observed that paternal iAs exposure caused glucose intolerance in the female offspring but not the male offspring.

In line with the sex-specific effects of paternal iAs exposure on glucose metabolism [26], *in utero* iAs exposure causes insulin resistance and mild glucose intolerance in female offspring but higher insulin sensitivity and unchanged glucose tolerance in male offspring in a rat model [19]. Maternal iAs exposure in mice also impairs glucose metabolism in offspring in a sex-specific manner [23]. Indeed, the offspring’s sex emerges as a critical modulator of the general intergenerational metabolic effects of many chemical or dietary exposures [27]. The underlying mechanisms for the sex differences are not completely understood.

Our previous study was limited to glucose metabolism. In the current study, we identified a robust dichotomous sex difference in F1 offspring in both glucose and lipid metabolism using a more prolonged paternal iAs treatment. We found that paternal iAs impaired glucose tolerance in F1 females but improved glucose tolerance in F1 males. In contrast, paternal iAs does not affect blood or liver lipid levels in F1 females but reduced lipid levels in F1 males. We systemically dissected the mechanisms underlying the sex differences in both glucose and lipid metabolism using ovariectomy and genetic mouse models with the tissue-specific knockout of estrogen receptors. Thus, the current study provides a framework for understanding sex differences in the intergenerational metabolic effects of chemical exposure.

## Results

### Paternal iAs exposure causes sex-specific effects on glucose metabolism in F1 offspring

**Fig. 1:**
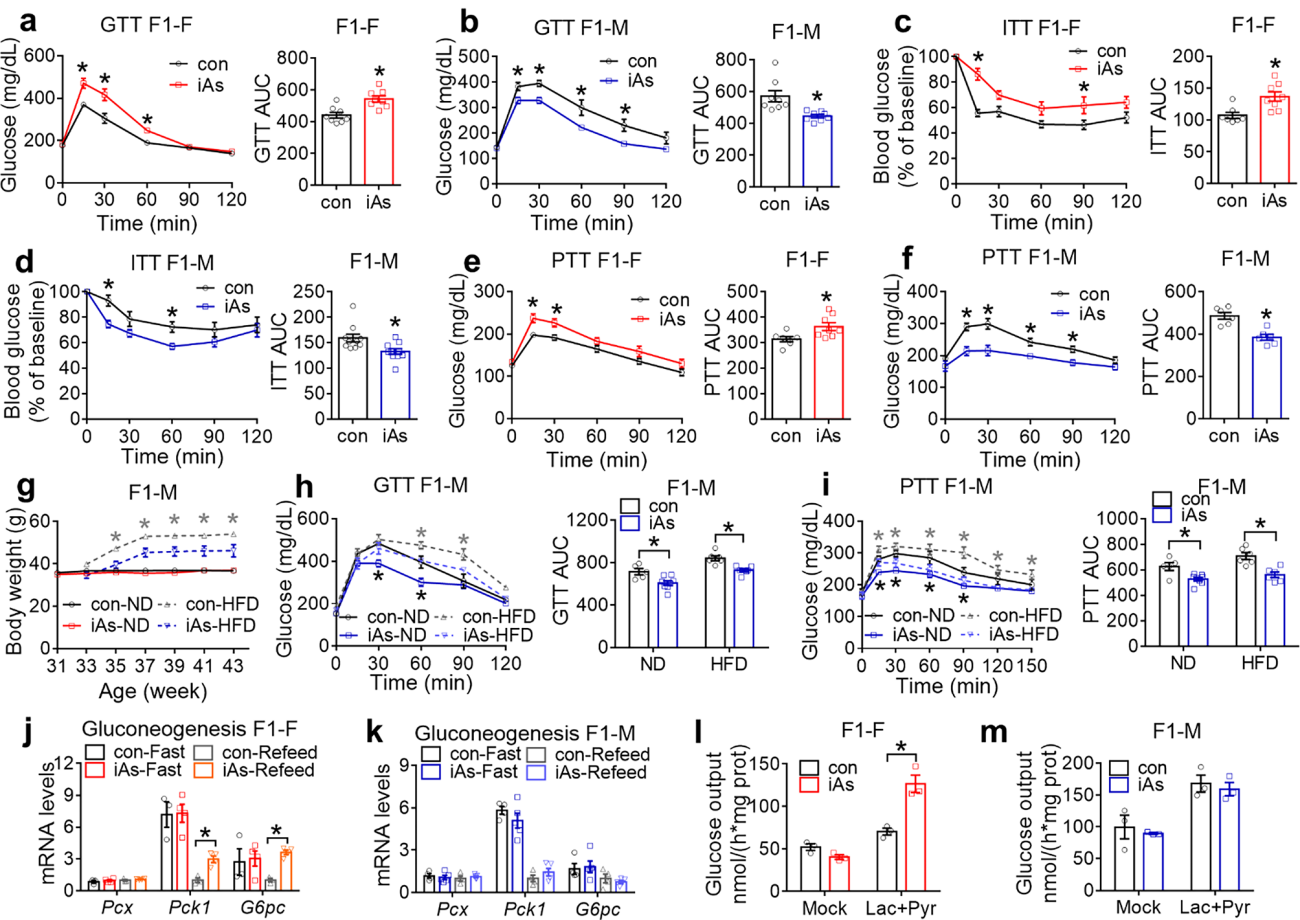
Sex difference in glucose metabolism in F1 offspring in response to paternal iAs. **(a)** Glucose tolerance test (GTT) and area under the curve (AUC) for control (con) or iAs F1 females (F1-F) at 12 weeks old, n = 8 mice. **(b)** GTT of F1 males (F1-M) at 12 weeks old, n = 8 mice. **(c)** Insulin tolerance test (ITT) of F1 females at 14 weeks old, n = 7 mice for con and n = 8 mice for iAs. **(d)** ITT of F1 males at 14 weeks old, n = 10 mice. **(e)** Pyruvate tolerance test (PTT) of F1 females at 20 weeks old, n = 7 mice for con and n = 8 mice for iAs. **(f)** PTT of F1 males at 20 weeks old, n = 6 mice. **(g)** Body weight of F1 males on a normal chow diet (ND) or a high-fat diet (HFD). HFD started at 33 weeks old. n = 5 mice for con-ND, n = 8 mice for iAs-ND, and n = 6 mice for con-HFD or iAs-HFD. **(h)** GTT of F1 males on the indicated diet at 41 weeks old, n = 5 mice for con-ND, n = 8 mice for iAs-ND, and n = 6 mice for con-HFD or iAs-HFD. **(i)** PTT of F1 males on the indicated diet at 43 weeks old, n = 5 mice for con-ND, n = 8 mice for iAs-ND, and n = 6 mice for con-HFD and iAs-HFD. **(j)** RT-qPCR analysis of gluconeogenic genes in the liver of F1 females under 7 h fasting (Fast) or 7 h refeeding (Refeed) conditions. The mean value in the con-Refeed group was set as 1. n = 3 mice for control groups and n = 4 mice for iAs groups. **(k)** RT-qPCR analysis of gluconeogenic genes in the liver of F1 males under 7 h fasting (Fast) or 7 h refed (Refeed) conditions, n = 4 mice for con groups and n = 5 mice for iAs groups. **(l)** Glucose output assay (GOP) in primary hepatocytes isolated from F1 females at 22 weeks old, n = 3 mice. **(m)** GOP in primary hepatocytes isolated from F1 males at 22 weeks old, n = 3 mice. Black or gray asterisks indicate significant differences between con and iAs groups on ND or HFD, respectively. Two-way ANOVA with Holm-Sidak method was used to analyze GTT, PTT, ITT, time-dependent body weight changes, gene expression changes, GOP, and 4-group AUC of kinetic metabolic tests. Two-sided t-test was used to analyze 2-group AUC of kinetic metabolic tests. Data are mean ± S.E.M. * *P* < 0.05

We focused on male-lineage exposure to minimize developmental effects from *in-utero* maternal exposure in the F1 offspring. We chose 250 ppb (parts per billion) iAs (250 µg arsenic element per liter) in drinking water for mouse studies after considering several factors. (1) As a drug, iAs can be used at 0.15 mg/kg daily or higher in humans [28], which corresponds to over 2 ppm (parts per million) in drinking water. (2) As an environmental chemical, the safety guideline for iAs in drinking water is below 10 ppb, as set by the World Health Organization and U.S. Environmental Protection Agency [29]. (3) Mice have a higher rate of iAs metabolism and clearance than humans [30]. Concentrations of iAs and metabolites in tissues in humans exposed to 0.22-2 ppm iAs in drinking water are comparable to those in mice exposed to 50 ppm iAs, which is more than a hundred times more than those in mice exposed to 1 ppm iAs in drinking water [30]. These results suggest that mice have over ten times higher iAs clearance rates than humans. Thus, the 250 ppb iAs exposure to mice is comparable to humans exposed to around or above 10 ppb. (4) Our previous studies have shown that 250 ppb iAs in drinking water is sufficient to cause inter- and trans-generational effects in F1 and F2 offspring, even in the absence of a robust effect in the F0 males [26]. Thus, we exposed male mice (F0) to 250 ppb iAs in drinking water for 6 weeks before mating with unexposed female mice to generate female and male F1 offspring (iAsF1-F and iAsF1-M, respectively). Unexposed male mice were bred simultaneously to generate female and male controls (conF1-F and conF1-M, respectively) (Supplementary Fig S1a). In line with our previous study with the 3-week paternal iAs exposure [26], 6-week paternal iAs exposure did not affect the litter size, birth weight, or bodyweight gain of the F1 offspring (Supplementary Fig S1b-e). All iAsF1 mice were born full-term. Adult iAsF1-F mice were glucose intolerant compared to conF1-F mice in the glucose tolerance test (GTT) (Fig. 1a). In contrast, adult iAsF1-M mice showed the opposite phenotype, with improved glucose tolerance compared to conF1-M mice (Fig. 1b). Considering that 3-week paternal iAs exposure did not improve glucose tolerance in iAsF1-M mice [26], these findings suggest that a more prolonged paternal iAs exposure causes more robust sex-specific effects in F1 offspring.

Glucose intolerance can arise from insufficient pancreatic insulin secretion or peripheral insulin resistance in the liver, adipose tissue, or muscle [31]. Blood insulin levels did not show significant differences in either sex between iAsF1 and conF1 at the baseline or at 15 min after intraperitoneal glucose injection (Supplementary Fig S1f-g). Indices of pancreatic β-cell functions did not differ significantly in either sex (Supplementary Fig S1h-i). Indices of insulin resistance showed differences consistent with the glucose tolerance phenotypes in both sexes (Supplementary Fig S1j-k), suggesting that insulin resistance is a major contributing factor to the sex disparity. Insulin tolerance test (ITT) further revealed inadequate responses to insulin in iAsF1-F mice but improved responses in iAsF1-M mice compared to their respective controls (Fig. 1c-d), suggesting that altered insulin sensitivity underlies the opposite glucose tolerance phenotypes in iAsF1-F mice vs. iAsF1-M mice.

Insulin suppresses hepatic glucose production, a major contributor to insulin-mediated control of glucose homeostasis. In the pyruvate tolerance test (PTT), iAsF1-F mice showed an elevated glucose excursion compared to conF1-F mice after a bolus injection of pyruvate, a gluconeogenic precursor (Fig. 1e), suggesting higher hepatic glucose output and impaired hepatic insulin sensitivity. In contrast, iAsF1-M mice displayed the opposite phenotype and showed a lower glucose excursion than conF1-M mice (Fig. 1f), suggesting impaired glucose output or hepatic insulin hypersensitivity in iAsF1-M mice compared to conF1-M mice. Since the high-fat diet (HFD) could lower insulin sensitivity and cause glucose intolerance, we wondered whether the phenotypes in F1 males caused by paternal iAs exposure could be potentiated or masked upon HFD. Although there was no change in food intake between iAsF1-M and conF1-M mice under a normal chow diet (ND) or high-fat diet (HFD) (Supplementary Fig S1l), the difference in energy metabolism between iAsF1-M and conF1-M mice was potentiated by HFD feeding, with iAsF1-M mice showing resistance to HFD-induced body weight gain (Fig. 1g and Supplementary Fig S1m), glucose intolerance (Fig. 1h), and pyruvate intolerance (Fig. 1i).

We focused on the ND groups for downstream molecular studies to avoid potential artifacts due to bodyweight differences between iAsF1-M and conF1-M on HFD. The expression of gluconeogenic genes, including Pck1 and G6pc, was upregulated in the liver of iAsF1-F mice vs. conF1-F mice in the refed condition (Fig. 1j), which was consistent with the glucose intolerance. In contrast, there was no significant change in the expression of these genes in iAsF1-M mice compared to conF1-M mice (Fig. 1k). In line with the gene expression analysis and the in vivo glucose phenotype, primary hepatocytes from iAsF1-F mice showed higher glucose output than conF1-F mice (Fig. 1l). In contrast, primary hepatocytes from iAsF1-M mice did not show changes in glucose output compared to conF1-M mice (Fig. 1m). These results suggest that the insulin resistance in the iAsF1-F liver likely depends on the long-lasting changes in hepatocytes. By comparison, the lower hepatic glucose output in the iAsF1-M mice vs. conF1-M mice may be due to transient changes that were lost in isolated primary hepatocytes. In summary, paternal iAs exposure causes opposite phenotypes in glucose metabolism and insulin sensitivity in female vs. male F1 offspring. The results pinpoint the liver as a major organ underlying this sex difference.

### Paternal iAs exposure causes sex-specific effects on lipid metabolism in F1 offspring

**Fig. 2:**
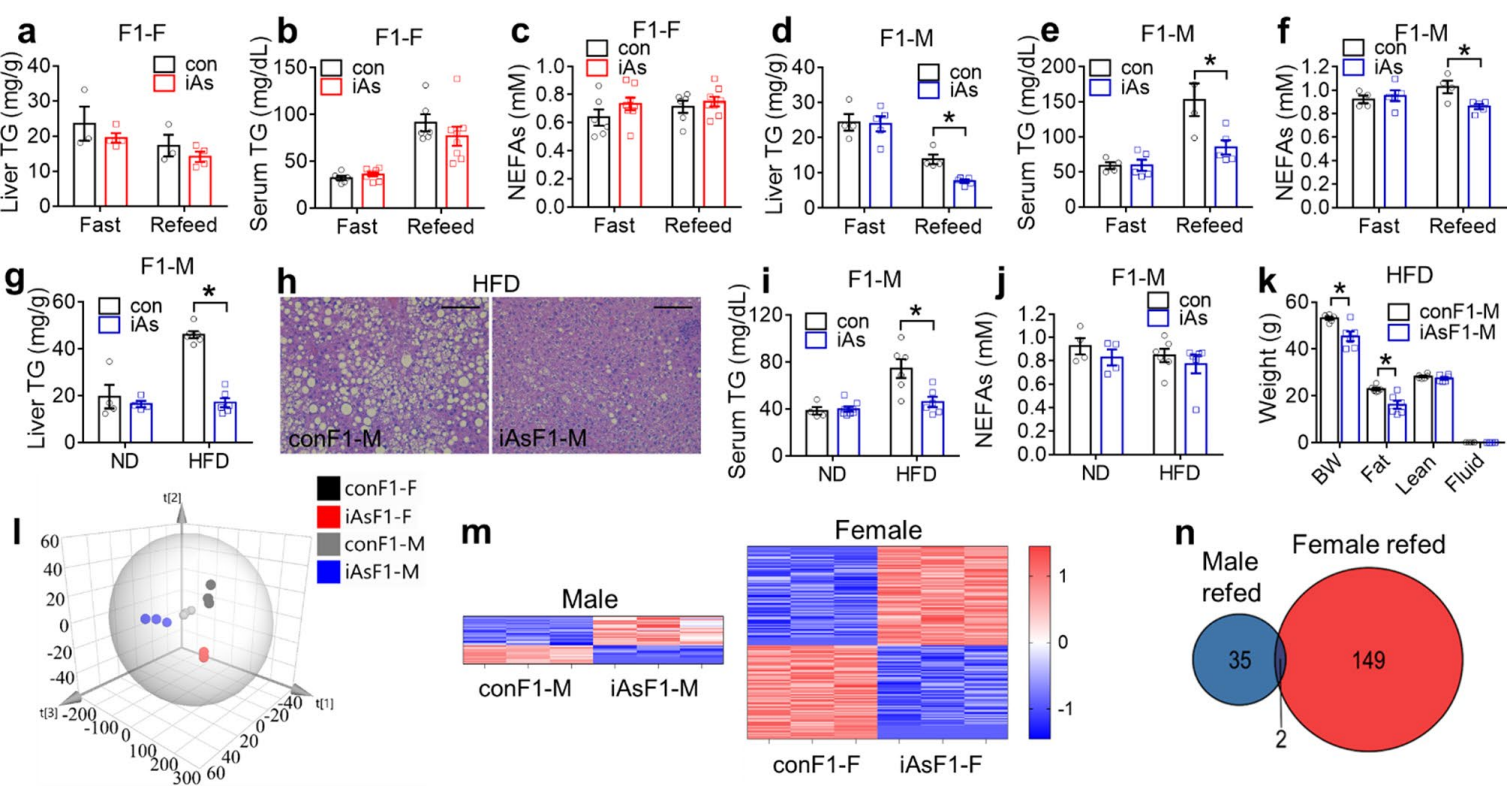
Sex difference of lipid metabolism for F1 offspring in response to paternal iAs. (**a**) Liver triglyceride (TG) contents in F1 females (F1-F), n = 3 mice for control (con) and n = 4 mice for iAs groups. (**b-c**) Serum TG and non-esterified fatty acids (NEFAs) levels in F1 females, n = 6 mice for control and n = 8 mice for iAs groups. (**d**) Liver TG contents in F1 males (F1-M), n = 4 mice for control and n = 5 mice for iAs groups. (**e-f**) Serum TG and NEFAs levels in F1 males, n = 4 mice for control and n = 5 mice for iAs groups. (**g**) Liver TG contents in F1 males at the fasting condition on the indicated diet, n = 4 mice for normal chow diet (ND) groups, and n = 6 mice for high-fat diet (HFD) groups. (**h**) Hematoxylin and eosin (H&E) staining of livers in F1 males on HFD. Scale bar, 100 μm. (**i-j**) Serum TG and NEFAs levels in F1 males, n = 4–8 mice for ND groups and n = 6 mice for HFD groups. (**k**) Magnetic resonance imaging (MRI) analysis of F1 males on HFD, n = 6 mice. **(l)** Partial least squares of discriminant analysis (PLS-DA) of the RNA-seq results of the livers in the conF1-F, iAsF1-F, conF1-M, and iAsF1-M groups in the refed condition, n = 3 mice. (**m**) Heat map of differentially expressed genes (DEGs) due to paternal iAs in either male or female F1 livers as identified by RNA-seq with q < 0.05 as cut-off. The color key shows Z-Score calculated as (FPKM-Mean)/SD. (**n**) Venn diagram of hepatic DEGs in iAsF1-F vs. conF1-F mice and DEGs in iAsF1-M vs. conF1-M mice in the refed condition, n = 3 mice. Two-way ANOVA with Holm-Sidak method was used to analyze liver TG levels, serum TG and NEFAs levels, and MRI data. Data are mean ± S.E.M. * *P* < 0.05 between conF1 and iAsF1 groups under the same conditions

Lipid metabolism is among the top enriched biological pathways by genes with sex-biased expression in the liver [32], and is known to play critical roles in insulin resistance [31]. Given the opposite glucose phenotypes between male and female iAsF1 offspring, we wonder whether there is a sex difference in lipid metabolism, which could be etiologically related to the glucose phenotype. Liver triglycerides (TG) content showed no difference between iAsF1-F and conF1-F mice (Fig. 2a and Supplementary Fig S2a). There was no change in blood lipid or ketone profiles in iAsF1-F mice compared to conF1-F mice (Fig. 2b-c and Supplementary Fig S2b). By comparison, iAsF1-M mice showed lower liver TG (Fig. 2d and Supplementary Fig S2c), lower blood TG (Fig. 2e), and lower blood non-esterified fatty acids (NEFAs) (Fig. 2f) during the refed condition compared to conF1-M mice, with no change in blood β-hydroxybutyrate (BHBA) (Supplementary Fig S2d). Such a male-specific lipid phenotype was further potentiated by HFD feeding even during the fasting condition (Fig. 2g-j). The hepatic TG content showed the most prominent change (Fig. 2g), with a drastic reduction in the size and number of lipid droplets in the iAsF1-M liver compared to conF1-M liver on HFD (Fig. 2h). The magnetic resonance imaging (MRI) showed that iAsF1-M mice had less fat mass than conF1-M mice on HFD, with normal lean mass (Fig. 2k). No obvious morphological change was observed in the white adipose tissue (WAT) between iAsF1-M and conF1-M on HFD (Supplementary Fig S2e). No prominent gene expression changes were found in WAT between iAsF1-M and conF1-M mice either (Supplementary Fig S2f-h), suggesting that changes in other tissues might be more mechanistically important. In summary, these findings demonstrated that paternal iAs exposure caused a prominent change in lipid metabolism in F1 males but not in F1 females.

**Fig. 3:**
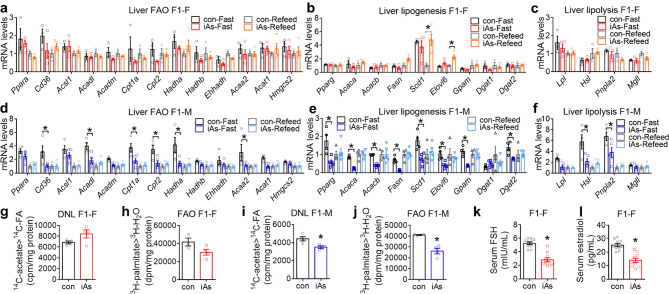
Paternal iAs alters hepatic lipid metabolic gene expression and sex hormones in F1 offspring. **(a-c)** RT-qPCR analysis of genes in fatty acid oxidation (FAO), lipogenesis, and lipolysis in the liver of F1 females (F1-F) after 7 h fasting or 7 h refed conditions, n = 3 mice for control groups and n = 4 for iAs groups. The mean value in the con-Refeed groups was set as 1. **(d-f)** RT-qPCR analysis of genes in FAO, lipogenesis, and lipolysis in the liver of F1 males (F1-M), n = 4 mice control groups and n = 5 for iAs groups. **(g)**^14^ C-acetate tracer analysis of de novo lipogenesis (DNL) in primary hepatocytes from F1 females at 22 weeks old, n = 4 mice. **(h)**^3^ H-palmitate tracer analysis of FAO in primary hepatocytes from F1 females at 22 weeks old, n = 3–4 mice. **(i)**^14^ C-acetate tracer analysis of DNL in primary hepatocytes from F1 males at 22 weeks old, n = 4 mice. **(j)**^3^ H-palmitate tracer analysis of FAO in primary hepatocytes from F1 males at 22 weeks old, n = 4 mice. **(k-l)** Serum follicle-stimulating hormone (FSH) and estradiol levels in F1 females, n = 10 mice. Two-way ANOVA with the Holm-Sidak method was used to analyze gene expression levels. Two-sided t-test was used to analyze isotope tracer assays, serum FSH levels, and serum estradiol levels. Data are mean ± S.E.M. * *P* < 0.05 between conF1 and iAsF1 groups under the same conditions

The altered body weight on HFD can cause secondary changes to liver lipid content. Therefore, we focused on the normal chow diet for further mechanistic analysis of the liver. RNA-seq analysis of the liver in the refed condition showed that the overall transcriptome patterns of the four groups (conF1-F, iAsF1-F, conF1-M, and iAsF1-M) were distinct from one another (Fig. 2l). The effects of paternal iAs exposure showed sex differences in the F1 offspring (Fig. 2m). Although the female dataset was from the 3-week iAs exposure while the male dataset was from the 6-week exposure, there were 151 differentially expressed genes (DEGs) in iAsF1-F vs. conF1-F but only 37 DEGs in iAsF1-M vs. conF1-M, with few overlapping DEGs between sexes (Fig. 2n). The different DEGs numbers and low DEGs overlap between sexes demonstrated different responses of F1 females and males to paternal iAs exposure. Gene ontology analysis did not identify significant enrichment for the DEGs in male mice, probably due to the small number of DEGs. RT-qPCR analyses confirmed that there were no widespread changes in lipid metabolic genes under either fasting or refed condition except for two upregulated genes involved in fatty acid synthesis in the iAsF1-F vs. conF1-F livers (Fig. 3a-c). The lack of major changes in lipid metabolic genes in iAsF1-F vs. conF1-F livers is in line with the overall minimal lipid metabolic phenotype in iAsF1-F vs. conF1-F mice (Fig. 2a-c). Although fewer DEGs were identified between iAsF1-M and conF1-M during the refed condition, the iAsF1-M liver exhibited prominent downregulation of genes involved in lipogenesis, fatty acid oxidation (FAO), and lipolysis in the fasting condition (Fig. 3d-f). Since downregulated FAO or lipolysis, at least by themselves, would not explain the lower lipid levels in the liver or blood, the downregulated lipogenesis is likely the primary cause of the lipid phenotype in iAsF1-M mice. In line with this notion, chronic HFD feeding in F1 males retained the lipid phenotype (Fig. 2g-j) and the differential expression of genes in lipogenesis (Supplementary Fig S3a) but diminished the difference for FAO and lipolysis genes (Supplementary Fig S3b-c). The blunted difference in FAO gene expression could be due to the activation of lipid-sensing transcriptional regulators in the presence of HFD [33, 34]. The prominent differences in gene expression in the fasting condition can contribute to the differential physiological responses to dietary nutrients upon refeeding, as mice undergo rhythmic fasting/refeeding cycles in normal diurnal physiology. In summary, the simultaneous downregulation of lipid anabolic and catabolic genes suggests a reduced overall capacity for lipid handling in iAsF1-M vs. conF1-M livers, including esterification, storage, lipolysis, and oxidation.

Compared to conF1-F, primary hepatocytes from iAsF1-F mice showed a trend of increased *de novo* lipogenesis (DNL) (Fig. 3g) and a trend of reduced FAO rate (Fig. 3h), although neither was statistically significant. Since upregulation of DNL flux and reciprocal downregulation of FAO flux is generally associated with selective insulin resistance [35] and is in line with FAO suppression by lipogenic intermediates [36], the trends of lipid metabolic fluxes in iAsF1-F vs. conF1-F hepatocytes are in keeping with the insulin resistance phenotype. In contrast, primary hepatocytes from iAsF1-M mice exhibited a lower flux rate in DNL (Fig. 3i) and FAO (Fig. 3j) than conF1-M. Acaca and Acacb (also known as ACC1 and ACC2) catalyze the first committed step of lipogenesis [37] and their inhibition can alter the expression of many genes in lipid synthesis and FAO [38]. Fasn and Elovl6, key lipogenic enzymes, can also regulate the expression of many lipogenic and FAO genes [39, 40]. Pnpla2 (also known as ATGL) catalyzes the first reaction of lipolysis [41], and its deficiency can lead to the widespread downregulation of FAO genes expression [42]. Thus, dysregulated expression of only a few key genes could explain the widespread gene expression changes across multiple lipid metabolic pathways, likely through altering substrate availability and lipid-sensing regulators. In summary, paternal iAs exposure did not drastically alter lipid metabolic fluxes in F1 female hepatocytes but robustly repressed DNL and FAO in F1 male hepatocytes. These results support disrupted lipid handling in iAsF1-M vs. conF1-M livers.

### Paternal iAs exposure disrupts reproductive hormones levels in F1 female offspring

In searching for the mechanisms underlying the sex-specific effects of paternal iAs exposure, we measured sex-related hormones in F1 offspring. Both the circulating follicle-stimulating hormone (FSH) level at the estrus phase and the circulating estrogen level at the proestrus phase were reduced in iAsF1-F vs. conF1-F mice (Fig. 3k-l). Estrogen is known to suppress liver glucose production and downregulate the expression of gluconeogenic genes such as G6pc and Pck1 [43–46]. Therefore, the reduced circulating estrogen in iAsF1-F mice may account for the glucose metabolism phenotype through impaired estrogen signaling in the liver, which would explain the sex difference for the glucose phenotype. The lack of lipid metabolic gene expression changes in iAsF1-F vs. conF1-F could also be attributable to estrogen. Since estrogen represses the expression of hepatic lipogenic genes [47–50], it could mask the repressive effects of paternal iAs in F1 females. By comparison, the low estrogen levels in F1 males allow the paternal iAs effects to be manifested. Thus, estrogen signaling could explain the sex differential effects of paternal iAs in both glucose and lipid metabolism.

### Gonadal hormones mediate the sex difference in F1 offspring

**Fig. 4:**
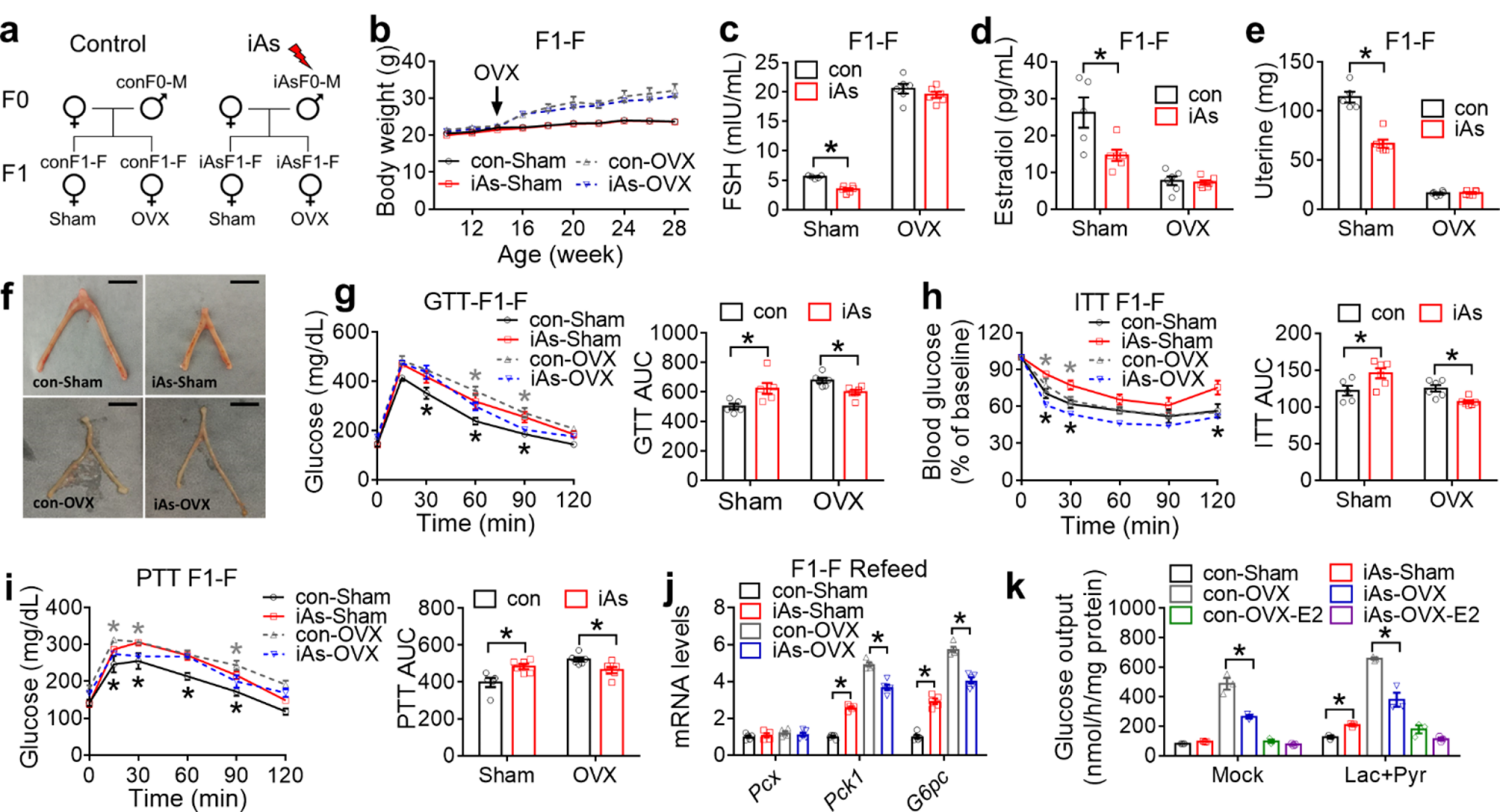
Sex hormones in F1 females mediate female-specific glucose phenotypes of paternal iAs. **(a)** Experimental scheme for F1 females (F1-F) with sham or ovariectomy (OVX) surgery at 14 weeks old. **(b)** Body weight, n = 5–6 mice. **(c-d)** Serum follicle-stimulating hormone (FSH) and estradiol levels in F1 females, n = 5–6 mice. **(e)** Uterine weight of F1 females after surgery, n = 5–6 mice. **(f)** Representative pictures of uterine from F1 females after sham and OVX. Scale bar, 5 mm. **(g)** Glucose tolerance test (GTT) and area under the curve (AUC) in F1 females at 22 weeks old after sham or OVX, n = 5–6 mice. Black or gray asterisks indicate significant differences between con and iAs groups after sham or OVX, respectively. **(h)** Insulin tolerance test (ITT) in F1 females at 24 weeks old after sham or OVX, n = 5–6 mice. **(i)** Pyruvate tolerance test (PTT) in F1 females at 20 weeks old after sham or OVX, n = 5–6 mice. **(j)** RT-qPCR analysis of gluconeogenic genes in the liver of F1 females in the fasting condition after sham or OVX, n = 5–6 mice. The mean value in the con-sham group was set as 1. **(k)** Glucose output (GOP) in primary hepatocytes from F1 females after sham or OVX at 22 weeks old, n = 3 for each group. Two-way ANOVA with the Holm-Sidak method was used to analyze GTT, PTT, ITT, 4-group AUC of kinetic metabolic tests, time-dependent body weight changes, gene expression levels, GOP, serum FSH and estradiol, and uterine weight. Data are mean ± S.E.M. * *P* < 0.05 between conF1 and iAsF1 groups under the same conditions

To test whether gonadal hormones are required for the sex-specific effects of paternal iAs exposure, we performed bilateral ovariectomy (OVX) or sham surgery in adult F1 females (Fig. 4a). OVX increased the body weight of conF1 and iAsF1 mice compared to sham mice, while no significant difference was observed between conF1-OVX and iAsF1-OVX mice (Fig. 4b). OVX changed blood FSH and estrogen levels compared to the sham groups, which blunted the difference between iAsF1-F and conF1-F mice as seen in the sham groups (Fig. 4c-d). Remarkably, iAsF1-F mice showed a drastic reduction in uterine weight and size compared to conF1-F mice (Fig. 4e-f). Since estrogen elevates the thickness of the uterine lining, the reduced uterine weight in iAsF1-F mice is consistent with their reduced estrogen levels compared to conF1-F. Importantly, the difference in uterine weight or size between iAsF1-F and conF1-F mice was blunted by OVX (Fig. 4e-f), demonstrating the role of gonadal hormones in this phenotype.

Interestingly, OVX flipped the effect of paternal iAs exposure on F1 females from impairing glucose tolerance to improving it (Fig. 4g), from insulin resistance to higher insulin sensitivity (Fig. 4h), and from increasing hepatic glucose production from pyruvate to suppressing it (Fig. 4i). Even though iAsF1-OVX mice showed unchanged serum insulin levels and HOMA-β compared to conF1-OVX mice (Supplementary Fig S4a-b), OVX flipped the effect of paternal iAs on HOMA-IR from elevating it to repressing it upon glucose challenge (Supplementary Fig S4c). OVX upregulated gluconeogenic genes in the livers of both conF1 and iAsF1 mice, compared to their respective sham groups. Importantly, the upregulated gluconeogenic genes Pck1 and G6pc in the refed condition caused by paternal iAs exposure were abolished by OVX (Fig. 4j). Fresh primary hepatocytes from iAsF1-F sham mice showed higher glucose output than conF1-F sham mice (Fig. 4k). Such an iAs effect was abolished by OVX, resulting in lower glucose output in hepatocytes from iAsF1-OVX mice compared to conF1-OVX mice (Fig. 4k). In addition, treating estradiol to hepatocytes from OVX mice masked the iAs effect (Fig. 4k). These results demonstrate that the reduced estrogen level in iAsF1-F vs. conF1-F mice accounts for the glucose phenotypic difference between the two groups. In summary, OVX in adult F1 female mice reversed the paternal iAs effects on glucose metabolism to resemble those in F1 male mice, demonstrating that gonadal hormones are required for the sex difference in glucose metabolism.

**Fig. 5:**
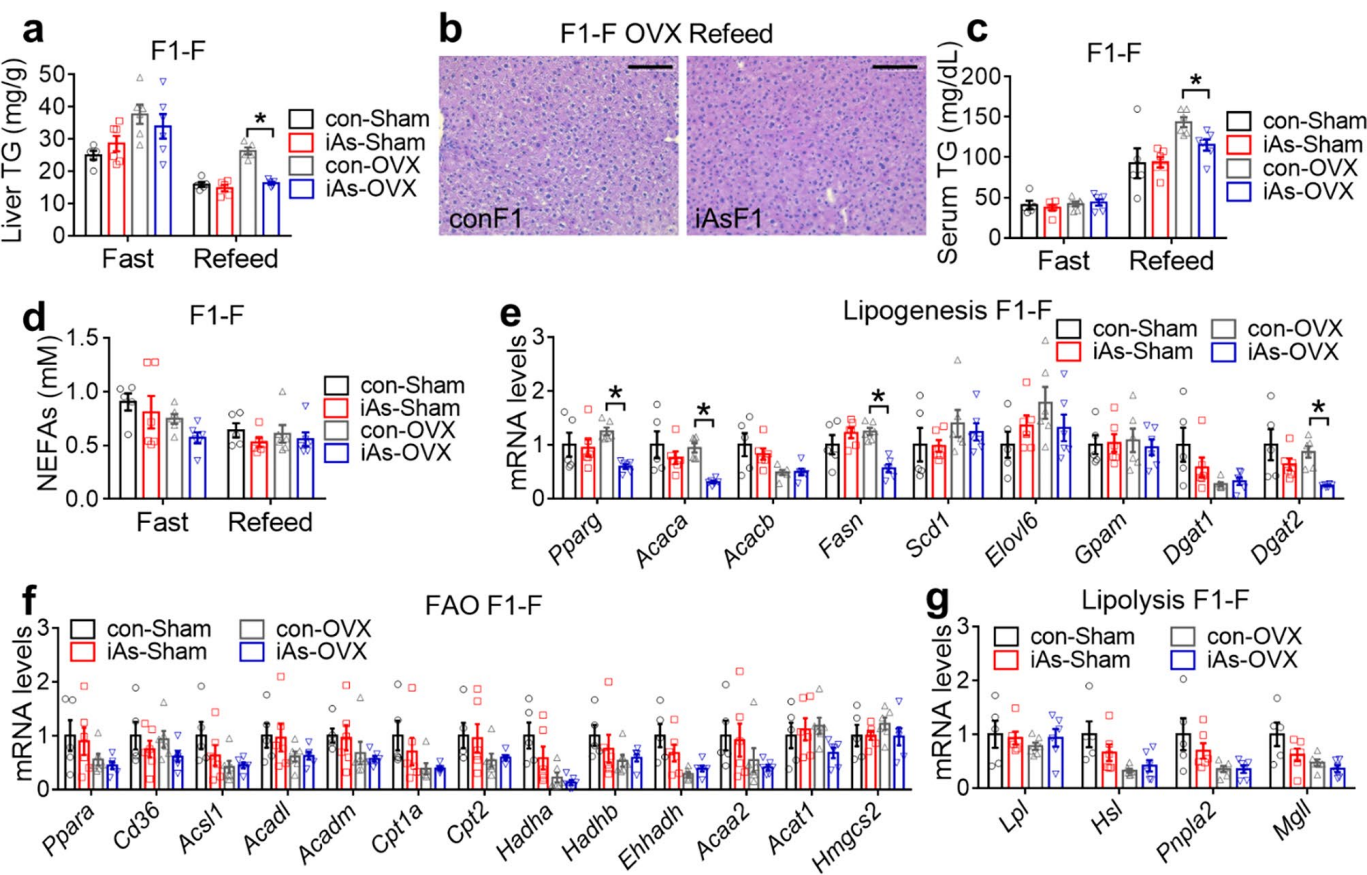
Gonad hormones in F1 females mask lipid phenotypes of paternal iAs. **(a)** Liver triglyceride (TG) contents in F1 females in the fasting condition after sham or OVX, n = 5–6 mice. **(b)** H&E staining of the liver in F1 females after sham or OVX. Scale bar, 100 μm. **(c-d)** Serum TG and non-esterified fatty acids (NEFAs) levels in F1 females after sham or OVX, n = 5–6 mice. **(e-g)** RT-qPCR analysis of genes in lipogenesis, fatty acid oxidation (FAO), and lipolysis in the liver of F1 females in the fasting condition after sham or OVX, n = 5–6 mice. The mean value in the con-sham group was set as 1. Two-way ANOVA with the Holm-Sidak method was used to analyze liver TG, serum TG, serum NEFAs, and gene expression levels. Data are mean ± S.E.M. * *P* < 0.05 between conF1 and iAsF1 groups under the same conditions

For lipid metabolism, the liver and serum TG levels in iAsF1-OVX mice were significantly lower than conF1-OVX mice in the refed condition (Fig. 5a-c), making them behave similarly to F1 males (Fig. 2d-e). No significant difference was identified in blood NEFAs levels between conF1-sham and iAsF1-sham mice or between conF1-OVX mice and iAsF1-OVX mice (Fig. 5d). iAsF1-OVX mice also showed downregulation of several lipogenic genes (Pparg, Acaca, Fasn, and Dgat2) in the liver in the fasting condition compared with conF1-F OVX mice, in contrast to the lack of differential expression in iAsF1-F vs. conF1-F livers in sham groups (Fig. 5e). OVX did not alter the effect of paternal iAs on the expression of genes in FAO and lipolysis (Fig. 5f-g). Collectively, these results suggest that OVX exposed the cryptic effects of paternal iAs exposure on lipid metabolism in iAsF1 females and made them resemble those in iAsF1 males. Thus, gonadal hormones in F1 females mask the lipid effects from paternal iAs exposure.

### Hepatic estrogen signaling is required for sex-specific effects of paternal iAs exposure

**Fig. 6:**
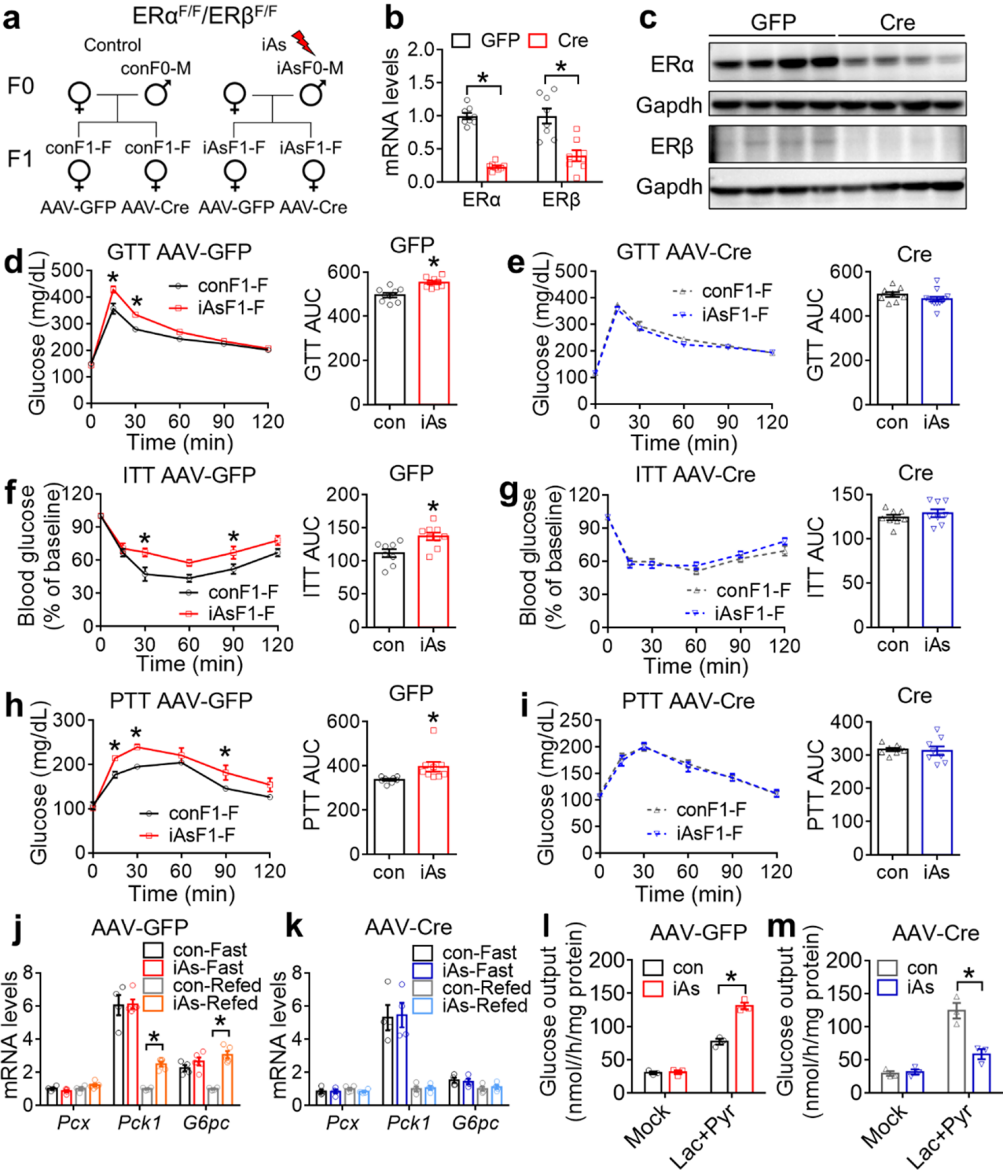
Liver ERα/β in F1 females is required for the glucose phenotypes of paternal iAs. **(a)** Experimental scheme for adult-onset liver-specific knockout of ERα/β in mice carrying double floxed ERα/β alleles through AAV injection at 19 weeks old. **(b)** RT-qPCR analysis of ERα/β in the liver of F1 females injected with AAV expressing GFP or Cre, n = 8 mice. **(c)** Western blot analysis of ERα and ERβ in the liver of F1 females injected with AAV-GFP or AAV-Cre, n = 4 mice. **(d-e)** Glucose tolerance test (GTT) and area under the curve (AUC) in F1 females (F1-F) at 27 weeks old, n = 8–12 mice per group. **(f-g)** Insulin tolerance test (ITT) in F1 females at 33 weeks old, n = 8–9 mice. **(h-i)** Pyruvate tolerance test (PTT) in F1 females at 31 weeks old, n = 8–9 mice. **(j-k)** RT-qPCR analysis of gluconeogenic genes in the liver of F1 females under fasting or refed condition. The mean value in the con-GFP or con-Cre groups was set as 1. n = 4–5 mice. **(l-m)** Glucose output (GOP) assay in primary hepatocytes from F1 females at 30 weeks old injected with AAV-GFP or AAV-Cre, n = 3 mice. Two-way ANOVA with the Holm-Sidak method was used to analyze GTT, PTT, ITT, gene expression levels, and GOP. Two-sided t-test was used to analyze 2-group AUC of kinetic metabolic tests. Data are mean ± S.E.M. * *P* < 0.05 between conF1 and iAsF1 groups under the same conditions

Given the changes in hepatic gene expression and hepatocyte-autonomous metabolic fluxes, we suspect that the liver is a major target of the female gonadal hormones in mediating the sex difference of the paternal iAs exposure. Estrogen binds to estrogen receptor α/β (ERα/β) and regulates gene transcription. We crossbred ERα floxed mice [51] with ERβ floxed mice [52] to generate homozygous double-floxed mice. We treated ERα/β double floxed male mice with 250 ppb iAs in drinking water for 6 weeks before mating them with non-exposed ERα/β double floxed female mice to obtain F1 offspring. Liver-specific double knockout of ERα/β in the adult F1-F offspring was then accomplished by tail vein injection of adeno-associated virus (AAV) expressing Cre recombinase under a hepatocyte-specific promoter (Fig. 6a). AAV expressing green fluorescent proteins (GFP) served as the wild-type control. Deletion of ERα/β in the liver was verified by RT-qPCR (Fig. 6b) and western blot (Fig. 6c). The seemingly less efficient deletion of ERβ compared to ERα was probably due to the lower baseline expression level of ERβ than ERα. No significant difference was observed in body weight between iAsF1-GFP and conF1-GFP (Supplementary Fig S5a) or between iAsF1-Cre and conF1-Cre female mice (Supplementary Fig S5b). ERα/β depletion abolished the phenotypic differences between iAsF1-F and conF1-F in glucose tolerance (Fig. 6d-e), insulin tolerance (Fig. 6f-g), and pyruvate tolerance (Fig. 6h-i). ERα/β depletion did not alter the iAs effect on insulin or HOMA-β but wiped off the iAs-induced elevation in HOMA-IR in the refed condition (Supplementary Fig S5c-h). The upregulation of gluconeogenic gene expression in iAsF1 vs. conF1 mice in the refed condition was also abolished upon depleting liver ERα/β (Fig. 6j-k). The higher glucose output in iAsF1 vs. conF1 primary hepatocytes was reversed in the absence of ERα/β (Fig. 6l-m), demonstrating an intricate interaction between paternal iAs and hepatic estrogen signaling. In summary, depletion of hepatic ERα/β in adult F1-F mice abolished the paternal iAs-induced glucose intolerance and insulin resistance, supporting that the female-specific glucose metabolic disruption by paternal iAs is due to the disrupted estrogen levels.

**Fig. 7:**
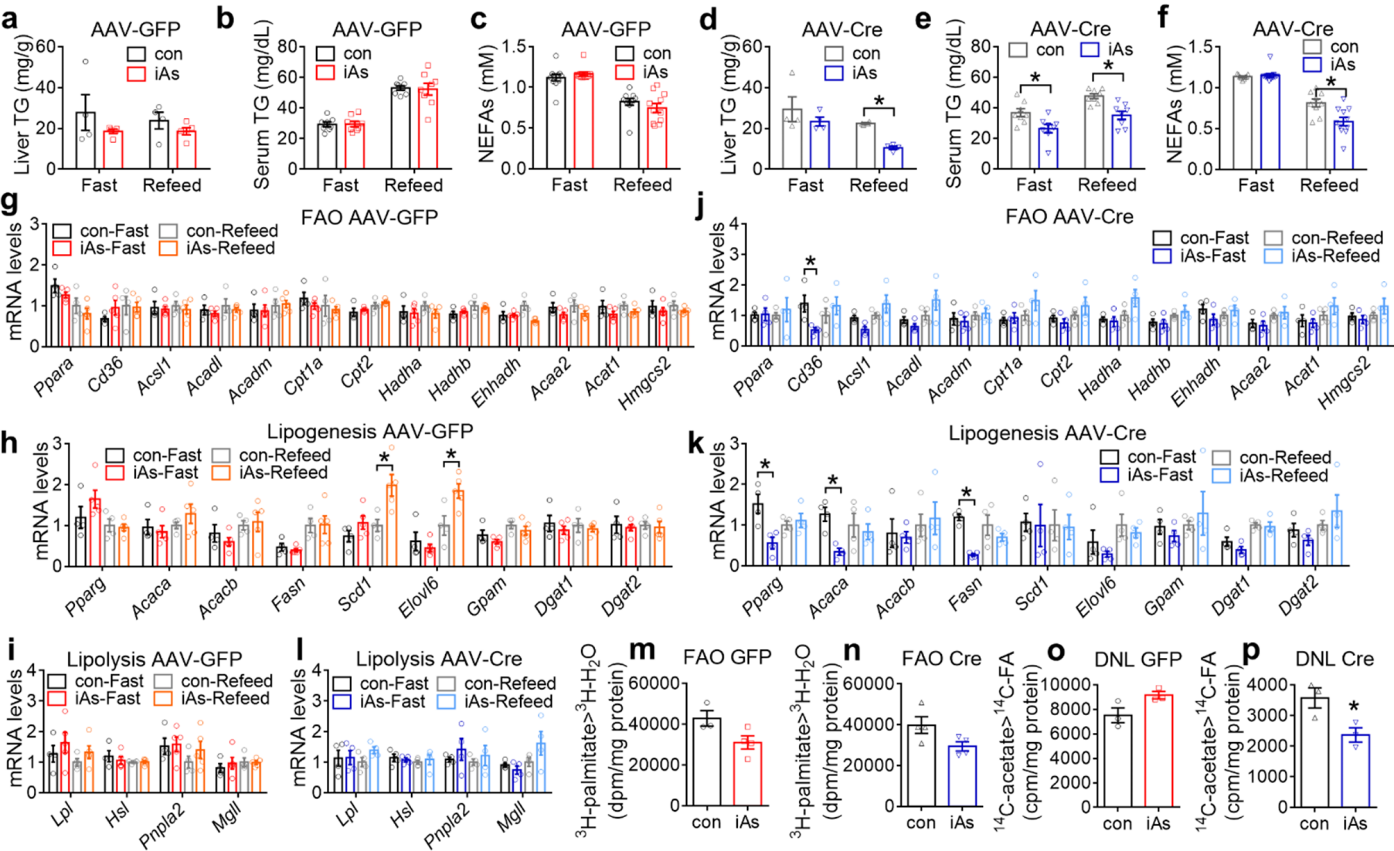
Liver ERα/β in F1 females counteracts the lipid metabolic changes of paternal iAs. **(a)** Liver triglyceride (TG) contents in F1 females injected with AAV-GFP, n = 4–5 mice. **(b)** Serum TG levels in F1 females injected with AAV-GFP, n = 8 mice. **(c)** Serum non-esterified fatty acids (NEFAs) levels in F1 females injected with AAV-GFP, n = 11 mice. **(d)** Liver TG contents in F1 females injected with AAV-Cre, n = 4 mice. **(e)** Serum TG levels in F1 females injected with AAV-Cre, n = 8 mice. **(f)** Serum NEFAs levels in F1 females injected with AAV-Cre, n = 9–11 mice. **(g-l)** RT-qPCR analysis of genes in fatty acid oxidation (FAO), lipogenesis, and lipolysis in fasting or refed condition in the liver of F1 females injected with AAV-GFP or AAV-Cre, n = 4–5 mice. The mean values in the con-GFP or con-Cre group were set as 1. **(m-n)**^3^ H-palmitate tracer analysis of FAO in primary hepatocytes from F1 female mice at 30 weeks old injected with AAV-GFP or AAV-Cre, n = 3–4 mice. **(o-p)**^14^ C-acetate tracer analysis of de novo lipogenesis (DNL) in primary hepatocytes from F1 female mice at 30 weeks old injected with AAV-GFP or AAV-Cre, n = 3 mice. Two-way ANOVA with the Holm-Sidak method was used to analyze liver TG, serum TG, serum NEFAs, and gene expression levels. Two-sided t-test was used to analyze isotope tracer assays. Data are mean ± S.E.M. * *P* < 0.05 between conF1 and iAsF1 groups under the same conditions

The liver-specific ERα/β knockout also modulated the lipid phenotype. Paternal iAs had a minimal impact on hepatic TG content, serum NEFAs level, or serum TG level in control F1 female mice injected with AAV-GFP (Fig. 7a-c), consistent with the previous results in WT F1-F mice (Fig. 2a-c). Interestingly, in the absence of liver ERα/β, iAsF1-F mice showed lower hepatic TG content (Fig. 7d and Supplementary Fig S6), serum TG level (Fig. 7e), and serum NEFAs level (Fig. 7f) compared to conF1-F mice in the fed condition, which resembles the lipid phenotypic changes in iAsF1-M mice compared to conF1-M mice (Fig. 2d-f). The expression of genes involved in FAO, lipogenesis, and lipolysis in the liver was minimally affected by paternal iAs exposure in the control mice injected with AAV-GFP (Fig. 7g-i), consistent with what was observed previously in WT F1-F mice (Fig. 3a-c). In the absence of liver ERα/β, the iAsF1-F liver showed downregulation for some of the genes in lipogenesis (Fig. 7j-l). In line with the gene expression results, ERα/β depletion did not affect the iAs effect on FAO fluxes in primary hepatocytes (Fig. 7m-n), but flipped the paternal iAs effect on lipogenesis from a trend of upregulation in iAsF1 vs. conF1 in WT mice to a significant downregulation in the absence of ERα/β (Fig. 7o-p). These findings support the causal role of hepatic lipogenesis in the lipid phenotype of iAsF1-M mice and demonstrate that hepatic ERα/β signaling in F1 females masked lipid metabolic phenotype from paternal iAs exposure, which accounts for the sex difference in lipid metabolism.

## Discussion

**Fig. 8:**
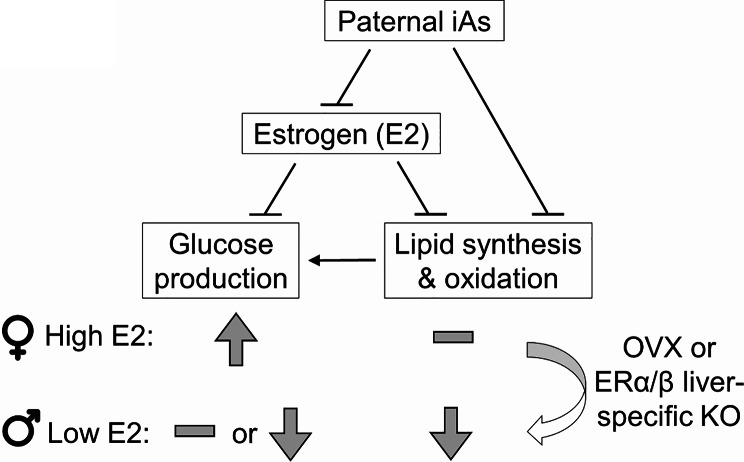
Working model. The sex difference in paternal iAs-induced glucose and lipid metabolic changes in F1 offspring

The current study is focused on dissecting sex differences of the intergenerational effect of paternal iAs exposure on glucose and lipid metabolism in F1 offspring. The data support the following working model (Fig. 8). On the one hand, paternal iAs exposure disrupts the reproductive hormones in F1 females, leading to reduced FSH secretion and blood estrogen levels. The low circulating estrogen level enhances hepatic glucose production by upregulating gluconeogenic gene expression through estrogen receptors. F1 male mice are less affected by this pathway because of their low baseline estrogen level. Thus, the low circulating estrogen in alliance with the hepatic ERα/β signaling explains the female-specific glucose phenotype in iAsF1 offspring. On the other hand, paternal iAs downregulates genes in lipogenesis and triglyceride synthesis in the liver of F1 male offspring, leading to reduced liver and blood TG or NEFAs, which may further repress lipolysis and fatty acid oxidation fluxes due to lack of substrates. The disrupted lipid handling can also contribute to impaired hepatic glucose production since lipid metabolism provides energy, precursors, and allosteric activators for glucose production. Such paternal iAs effects on lipid metabolism are only manifested in F1 males because the high estrogen levels in F1 females suppress hepatic lipid metabolism through hepatic estrogen receptors, which masks the paternal iAs effect and explains the male-specific lipid phenotype (Fig. 8).

This working model is supported by several lines of evidence in the current study and the literature. (1) Paternal iAs caused transcriptional changes in genes involved in gluconeogenesis and lipid metabolism in F1 adults, respectively. (2) Paternal iAs reduced FSH and estrogen levels and the uterine size in F1 female offspring. (3) Ovariectomy of F1 females reversed the paternal iAs-induced glucose phenotype and exposed the lipid phenotype. (4) Hepatocytes from adult F1 offspring display cell-autonomous changes in glucose metabolism and lipid metabolism in females and males, respectively. (5) Liver-specific deletion of ERα/β in adult F1 females abolished the paternal iAs-induced glucose phenotype but exposed the lipid phenotype. (6) Estrogen is known to protect females from insulin resistance and glucose intolerance. Estrogen treatment represses hepatic glucose production, downregulates the hepatic expression of G6pc and Pck1, and rescues OVX-induced glucose intolerance [43–46]. (7) Estrogen represses the hepatic expression of lipogenic genes and suppresses hepatic triglyceride accumulation [47–50].

The liver is likely the ultimate mediator of the sex-specific effects of paternal iAs exposure on both the glucose metabolic phenotype in F1 females and the lipid metabolic phenotype in F1 males because (1) the liver shows the most prominent changes in metabolic genes expressions among metabolic tissues we tested; (2) isolated primary hepatocytes from F1 offspring display sex-specific changes in glucose production and lipid metabolic fluxes consistent with metabolic gene expression changes; (3) liver-specific deletion of ERα/β in F1 females abolishes the sex differences in paternal iAs-induced metabolic effects. In line with these data, the liver is among the most responsive tissue to estrogen [53]. Estradiol treatment inhibits the expression of multiple lipid metabolic genes in the liver of obese ob/ob mice [54]. ERα is the predominant form of ER in the liver, while ERβ is expressed at a low level in the liver, with its function unclear. For OVX female mice lacking ERα, estradiol treatment failed to maintain VLDL production, limit diacylglycerol accumulation, or improve insulin sensitivity [55]. Therefore, it is possible that the effect of ERα/β double deletion in the liver in our study is mediated predominantly by ERα.

While multiple sex- or reproduction-related hormones could contribute to the sex difference, we chose to focus on estrogen and estrogen receptors in the current study in the spirit of Occam’s razor, considering that all the available data support the critical role of estrogen signaling in the sex differences in F1 offspring. The essential role of hepatic ERα/β in the intergenerational effects of iAs is in line with our previous finding that paternal iAs exposure lowered AKT-mediated inhibitory phosphorylation of FoxO1 at S256 in the liver of F1 females and upregulated Foxo1 target genes in gluconeogenesis [26]. ERs and FoxOs have been shown to interact with each other and counteract the function of each other [46, 56–59]. On one hand, ER promotes FoxO phosphorylation and represses FoxO-mediated transactivation [46, 56]. On the other hand, FoxO can co-localize with ER on the chromatin and serve as a corepressor to inhibit ER-mediated transcriptional activity [57–59].

Maternal exposure to 10 ppb or 42.5 ppm iAs in drinking water from gestational day 10 to birth increased body weight, body fat, and glucose intolerance in F1 female mice [22]. Another study showed that maternal exposure to 3 ppm iAs from prenatal stages to 4 months old damaged pancreatic β cells, reduced insulin secretion, and impaired glucose tolerance in F1 female rats [18]. Another study showed that continuous exposure to 50 ppm iAs in drinking water from gestational day 1 to 8 weeks old caused glucose intolerance in F1 female rats but unchanged glucose tolerance in F1 male rats [19]. Maternal exposure (E5 to birth) or continuous in utero and postnatal exposure (from E5 to 13 weeks old) to 100 ppb iAs increased body weight and hepatic triglyceride content, associated with insulin resistance, hyperglycemia, and upregulated hepatic genes involved in fatty acid synthesis and triglyceride packing in F1 male mice [21]. Simultaneous maternal and paternal exposure plus postnatal exposure to 100 ppb or 1000 ppb iAs (from 7 days before mating) caused fasting hyperglycemia and insulin resistance in F1 male mice but not in F1 female mice [23]. Likewise, simultaneously maternal and paternal exposure plus postnatal exposure to 3 ppm iAs (10 days before mating to 4 months old) increased lipid oxidation and the degradation of membrane lipids, which was more evident in F1 male rats than F1 female rats [24]. Thus, maternal iAs causes insulin resistance and glucose intolerance in F1 female offspring, in line with the paternal iAs effects we observed. However, paternal iAs increases insulin sensitivity and glucose tolerance in F1 male mice, opposite to the maternal iAs effects. In addition, maternal iAs or simultaneous maternal and paternal iAs exposure causes lipid accumulation in F1 male offspring, which is also opposite to the paternal iAs effect in reducing blood and liver TG levels in F1 male mice. Therefore, there are similarities and differences in the paternal and maternal iAs effects on glucose and lipid metabolism in F1 offspring, suggesting different underlying mechanisms.

There are several limitations in the current study. (1) We did not focus on dissecting mechanisms underpinning epigenetic inheritance. It remains an open question how paternal iAs exposure decreases blood FSH and estradiol levels in adult F1 female offspring and how paternal iAs exposure affects lipid metabolism in adult F1 male offspring. iAs was found to alter DNA methylation in vitro [60] and in vivo [61–64]. It remains unclear how iAs would alter DNA methylation in a loci-specific manner and, more importantly, to what degree DNA methylation changes contribute to the endocrine and metabolic changes in the offspring. In addition to DNA methylation, other epigenetic mechanisms, including histone/protamine modifications or noncoding RNAs, could all play a role in intergenerational inheritance. The interplay between estrogen signaling or lipogenesis and the epigenome is particularly intriguing. Estrogen receptors and key lipogenic enzyme genes might interact with epigenome reader proteins in orchestrating the chromatin remodeling events and gene expression regulation during fasting/refeeding cycles, which collectively account for the intergenerational effects in a sex-specific manner. (2) We only tested the paternal effects of iAs because the maternal effects or prenatal exposure have been studied in animals and could be complicated by developmental disruptions in addition to the epigenetic effects [14–24]. (3) We only studied animals. (4) We only tested one dosage of iAs.

There are a few human studies for prenatal iAs exposure in cancer, heart, pulmonary, and renal disease in humans [65]. Although the paternal and maternal effects are usually mixed with each other in these human studies, a common finding about early-life iAs exposure in human studies is the F1 offspring sex plays a critical role in the intergenerational effects [16]. There is no human study yet on prenatal or early-life iAs exposure in diabetes or metabolic disorders. Our results suggest that paternal effects alone warrant future investigations in human populations. In particular, we revealed that paternal iAs exposure generates sex-specific effects in glucose and lipid metabolism in the F1 offspring, which is attributable to the circulating estrogen levels and the hepatic estrogen receptor signaling. These findings provide mechanistic insights into sex differences in intergenerational long-term gene-environment interactions.

## Conclusions

Paternal iAs exposure caused sex disparity in glucose and lipid metabolism in F1 offspring. iAsF1-F showed glucose intolerance and insulin resistance with almost no change in blood lipid profiles while iAsF1-M showed improved glucose tolerance and insulin sensitivity with decreased lipid metabolic profiles. Ovariectomy or liver-specific knockout of estrogen receptor α/β made F1 females resemble F1 males in their metabolic responses to paternal iAs exposure. Therefore, disrupted reproductive hormone secretion in alliance with hepatic estrogen signaling accounts for the sex-specific intergenerational effects of paternal iAs exposure.

## Methods

### Animal

All the mice used in this study were of the C57BL/6 genetic background. ERα floxed mice were obtained from Dr. Sohaib A Khan [51]. ERβ floxed mice were obtained from JAX (032174) [52]. Sodium arsenite (NaAsO2, Sigma-Aldrich) was dissolved in Milli-Q water to make drinking water containing 250 ppb iAs (3.225 µM NaAsO2). After weaning, C57BL/6 WT male mice were treated with 250 ppb iAs in drinking water (iAsF0) or normal water (conF0) for 6 weeks and then bred with 15-week-old, non-exposed females for 3 days in cages with only normal water (without iAs), to generate iAsF1 or conF1 mice, respectively. F0 females and all F1 mice drank normal water without iAs all the time (Supplemental Fig S1a). The iAs exposure experiment was conducted in a designated chamber for environmental toxicity study. To minimize litter bias, in vivo metabolic analyses were conducted on F1 cohorts composed of more than 5 litters of distinct breeding pairs, with 1 or 2 mice randomly selected from each litter. Primary hepatocyte assays used F1 mice from 3 or 4 litters, with 1 mouse randomly selected from each litter. All F1 mice were group-housed (3–4 mice per cage) in a pathogen-free animal facility with standard 12 h light and 12 h dark cycles (7 am light-on and 7 pm light-off), at 22-25^o^C ambient temperature and 40–70% humidity condition with free access to water and food. All mice were fed with a normal chow diet (ND, LabDiet, D5015) except those indicated with “HFD” in the figures. ND consists of 20 kcal% protein, 26 cal% fat, and 54 kcal% carbohydrates with a total of 3.1 kcal/g energy density. HFD (Research Diets, D12492) consists of 20 kcal% protein, 60 cal% fat, and 20 kcal% carbohydrates with a total of 5.21 kcal/g energy density. The age of each experiment was indicated in figure legends. There was no specific reason for running a specific analysis at a particular age. We allow intervals of several weeks in-between bleeding to allow mice to recover from the stress. Tissues were harvested in the late afternoon within one hour for all cohorts after 7 h fasting or 7 h refed condition. All animal experiments were approved by the Institutional Animal Care and Use Committee (IACUC) at Baylor College of Medicine.

For the OVX experiment, mice were anesthetized with inhaled isoflurane, and eye ointment was applied to prevent corneal drying and damage. Hair in the flank area was shaved, and the skin was disinfected. Then, we incised the skin and musculature on one side, carefully pulled the ovarian fat pad out of the incision, clamped the region below the ovary, tightly made two knots, and finally removed the ovary. The ovarian fat pad was reposited, and the musculature and skin were sutured. The ovary on the other side was also excised using the same method. Meanwhile, bilateral OVX sham surgery was made for the other mice as controls. Mice after surgeries were observed daily for any inflammatory signs and painful behaviors in the following 7 days. For AAV injection, adult ERα^Flox/Flox^/ERβ^Flox/Flox^ F1 offspring from the control or iAs groups were injected with 200 µL AAV8-TBG-Cre at a concentration of 1.5 × 10^11^ genome copies/mL through the tail vein to obtain liver-specific double knockout of ERα/β, while AAV8-TBG-GFP served as the wild-type controls. AAV8-TBG-Cre and AAV8-TBG-GFP viral vectors were generated at the Baylor College of Medicine Gene Vector Core. Littermate controls were used for OVX experiments (sham vs. OVX) and virus injections (AAV-GFP vs. AAV-Cre). OVX and AAV injections were performed on two independent mouse cohorts. For MRI, mice were anesthetized with isoflurane and imaged on a Bruker Biospin 7.0T PharmaScan (Bruker Biospin, Billerica, MA, USA). Cross-sectional imaging modalities provide maps of pixels allowing for the in vivo measurement of lean mass, fat mass, and fluid.

**Kinetic metabolic testing** in vivo.

For ITT, GTT, or PTT, mice were handled daily for about a week before the test so that the mice were adapted to handling to minimize stress. On the day of kinetic metabolic testing, mice were subjected to fasting for 4 h for ITT, 6 h for GTT, or 8 h for PTT, followed by intraperitoneal injection of 0.75 mIU/kg insulin (Humulin, Eli Lily), 2 g/kg glucose (D-(+)-Glucose, Sigma-Aldrich), or 2 g/kg pyruvate (Sigma-Aldrich) in unrestrained conscious mice. At 0, 15, 30, 60, 90, and 120 min after intraperitoneal injection, blood was collected from the tail vein and was used to monitor glucose levels using a glucometer (OneTouch Ultra 2, Lifescan). The area under the curve (AUC) of glucose excursions was calculated for GTT, ITT, and PTT analysis by the trapezoidal rule as described previously [66].

### Blood biochemistry and ELISA

Microvette CB300 capillary blood collection tubes were used to collect blood samples from the tail vein. After centrifugation at 400 g for 15 min at 4 ℃, the supernate was transferred into a new tube and stored at -80 ℃ for further detection. Triglyceride Liquicolor Test (Stanbio Laboratory, Boerne, TX, USA), Non-Esterified Fatty Acid Fluorometric Assay Kit (Cayman Chemical, Ann Arbor, MI, USA), β-Hydroxybutyrate LiquiColor (Stanbio Laboratory, Boerne, TX, USA) were used to measure the TG, NEFAs, and BHBA levels in the serum following the manufacturer’s instruction. Blood samples collected at the conditions of fasting/refeeding or baseline/glucose challenge (15 min after 2 g/kg glucose injection) were used to measure insulin levels using an ultrasensitive mouse insulin ELISA Kit (#90,080, Crystal Chem, Inc., Chicago, IL). Blood glucose level and insulin level were used to calculate HOMA-IR and HOMA-beta indexes according to the following mathematical equations: HOMA-IR = (Glucose * Insulin) / 405 and HOMA-beta = (360 * Insulin) / (Glucose − 63) * 100%, in which the units of glucose level and insulin level were mg/dL and µU/mL, respectively. HOMA-IR and HOMA-beta were used to evaluate insulin resistance and beta-cell function. Blood samples collected at the proestrus phase were used to determine serum estrogen levels using a 17β Estradiol ELISA Kit (ab108667, Abcam, Cambridge, MA, USA). Blood samples collected at the estrus phase were used to detect serum FSH levels using an FSH ELISA Kit (Enzo Life Science, Inc., Farmingdale, NY, USA).

### RT-qPCR, western blot, and histology

Liver or visceral white adipose tissue samples for all the mice were collected from the same location and immediately stored in liquid nitrogen. Total RNA was extracted using Trizol reagent (Life Technologies) in combination with the RNeasy Mini Kit (Qiagen, Hilden, Germany). The quality and concentration of total RNA were checked on Nanodrop Microvolume UV-Vis Spectrophotometer (Thermo Fisher). Reverse transcription of an equal amount of total RNA from each sample was performed using a High-Capacity cDNA Reverse Transcription Kit (Life Technologies), and qPCR was conducted with SYBR Green Master Mix (Abcam) on Quant Studio 6 Instrument (Thermo Fisher). The sequences of primers used in the qPCR are shown in Supplementary Table S1. Standard curves were generated by series dilution of pooled RNA samples. The mRNA expression level of each gene was quantified with a standard curve and normalized to housing gene 18 S. For western blot, tissues were lysed in RIPA buffer containing 1 mM PMSF, 1mM DTT, and protease inhibitor. Total protein concentration in the lysate was detected using BCA Protein Assay Kit (Fisher Scientific), and equivalent protein was resolved by Tris-glycine SDS-PAGE, transferred to PVDF membrane, and blotted with antibodies against ERα (EMD Millipore 06-935), ERβ (Santa Cruz Biotechnology SC-390243X), and GAPDH (Cell Signaling Technology CST2118). For histology studies, fresh liver and visceral white adipose tissues were collected, fixed in 10% formalin for 24 h, and embedded in paraffin. Five µm sections were cut and stained with hematoxylin and eosin (H&E) and examined by microscopy (Leica, Wetzlar, Germany).

### RNA-seq

For RNA-seq, total RNA samples were used to prepare the DNA library and were sequenced on the Illumina Hiseq 2000/2500 platform using paired-end sequencing method. The raw sequencing data were filtered to remove low-quality reads containing sequencing adapter, more than 20% low-score base, or more than 5% unknown base. The clean reads were aligned to the mouse genome assembly GRCm38 using HISAT2. DEGs between the conF1-F and iAsF1-F mice or between conF1-M and iAsF1-M mice were identified using Deseq2 with *q* < 0.05. Partial least squares of discriminant analysis (PLS-DA) of the RNA-seq results of the livers in the conF1-F, iAsF1-F, conF1-M, and iAsF1-M groups in the refed condition was performed using SIMCA-P 13.0 software (Umetrics, Umea, Sweden).

### Primary hepatocytes isolation and glucose output assay

Primary hepatocytes were isolated from adult F1 female and male mice using the modified two-step perfusion method [26]. Perfusion buffer (HBSS containing 500 μm ethylene glycol tetraacetic acid and 50 µg/mL heparin) and digestion buffer (HBSS containing 0.5 mg/mL collagenase and 0.04 mg/mL trypsin inhibitor) was successively perfused into the liver under anesthesia through a catheter in the inferior vena cava. Cells were separated from liver tissue using cell scrapers and passed through a 70 μm cell strainer. Isolated cells were washed in Dulbecco′s Modified Eagle′s Medium (DMEM) and purified in the mixture of DMEM and 45% Percoll (Amersham Biosciences AB, Piscataway, NJ) using the density-gradient centrifugation method. Primary hepatocytes were seeded in collagen-coated plates with DMEM containing 10% FBS and 1% penicillin-streptomycin for about 4 h to allow attachment. For glucose output assay, primary hepatocytes were starved in glucose output media (4.7 mM KCl, 118 mM NaCl, 1.2 mM CaCl_2_, 1.2 mM MgSO_4_, 20 mM NaCO_3_,1.2 mM KH_2_PO_4_, 20 mM HEPES, and pH 7.4) for 1 h immediately after attachment, followed by treatment with substrates containing 1 mM pyruvate and 10 mM lactate for 2 h. Glucose release into the output media was measured using the Hexokinase Liquid Reagents Kit (Fisher Scientific G7517120) and was normalized to the total protein content of the cell lysates.

### DNL and FAO assay in vitro

For lipogenesis assay, primary hepatocytes were treated with DMEM containing 1 µCi/mL [1,2-^14^ C] -acetate, 10 mM cold acetate, 0.1 µM insulin, and 1 mM pyruvate for 2 h after attachment. The cells were then washed 3 times with PBS, detached from wells using ethanol and KOH mixture (v/v, 1:1), heated at 70 °C for 2 h, and dissociated in 5 M H_2_SO_4_. Organic metabolites were extracted 3 times from the lysates using hexane and mixed with a scintillation cocktail. The newly synthesized ^14^ C-FA in the organic metabolites was determined by a scintillation counter LS6500 (Beckman, Fullerton, CA, USA) and normalized to the total protein content in the cell lysate. For FAO assay, primary hepatocytes were washed 3 times with PBS without Ca and Mg and incubated in PBS containing 0.6 µCi/mL [9,10-^3^ H(N)]-palmitate conjugated on BSA and carnitine for 2 h. The incubation solution was treated with 10% trichloroacetic acid, neutralized with 6 M NaOH, and flowed through ion-exchange columns (DOWEX 1 × 4-400) to separate ^3^ H-H_2_O. The newly generated ^3^ H-H_2_O was counted in a scintillation counter and normalized to the total protein content in the cell lysate.

### Statistical analysis

No statistical methods were utilized to predetermine sample sizes. Instead, sample sizes were determined based on previous publications [26] and indicated for each experiment in the figure legends. To minimize litter bias, we generate each F1 cohort by randomly selecting F1 mice from more than 5 litters of different F0 breeding pairs, with 1 or 2 mice from each litter, for all in vivo experiments. For the ex vivo primary hepatocyte analysis, we used F1 mice from 3 or 4 litters, with 1 mouse from each litter. All statistical analyses and S.E.M. calculations were based on the individual mouse. Multiple group comparisons were analyzed using two-way ANOVA with Holm-Sidak method in the GraphPad Prism 9.0, including multi-timepoint GTT, PTT, and ITT, 4-group AUC of kinetic metabolic tests, expressional gene levels, time-dependent body weight changes, glucose output, and blood parameters. Comparisons between two groups were analyzed using a two-sided *t*-test, including 2-group AUC of kinetic metabolic tests, litter size, birth weight, and isotope tracer assays.

### Electronic supplementary material

Below is the link to the electronic supplementary material.

Supplementary Material 1

## Data Availability

RNA-seq datasets for F1 female and male mice were deposited in Gene Expression Omnibus (GSE154130) and (GSE205355), respectively. The review token for GSE205355 is avmpkooqrhqvtgn.
