# Supplementary material for: Sex differences in paternal arsenic-induced intergenerational metabolic effects are mediated by estrogen
Source: Cell Biosci. 2023 Sep 10;13:165. doi: 10.1186/s13578-023-01121-4 (PMC10493026; doi:10.1186/s13578-023-01121-4)
Supplement: Supplementary file 1 — Supplementary Material 1 [file 13578_2023_1121_MOESM1_ESM.docx]

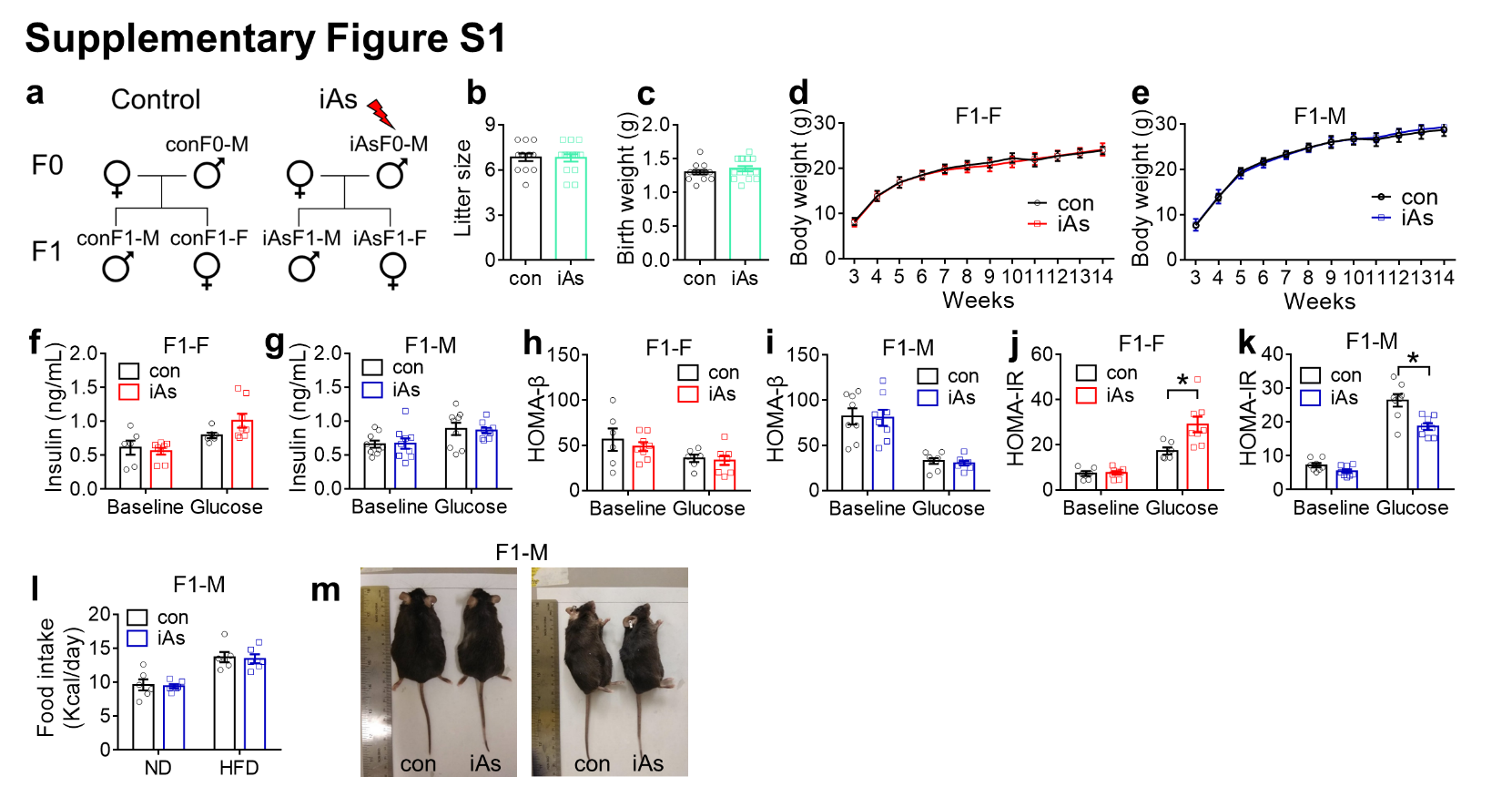


**Supplementary Figure S1. Body weight and blood parameters of F1 offspring.**

**(a)** Animal experimental scheme. **(b-c)** Litter size and birth weight of the F1 offspring, n = 13 litters for control (con) and n = 15 litters for iAs. **(d)** Body weight of F1 females (F1-F), n = 10 mice. **(e)** Body weight of F1 males (F1-M), n = 10 mice. **(f)** Serum insulin levels of F1 females at 15 weeks old, n = 6 mice for control and n = 8 mice for iAs groups. **(g)** Serum insulin levels of F1 males at 15 weeks old, n = 9 mice. **(h)** HOMA-β of F1 females, n = 6 mice for control and n = 8 mice for iAs groups. **(i)** HOMA-β of F1 males, n = 8 mice per group. **(j)** HOMA-IR of F1 females, n = 6 mice for control and n = 8 mice for iAs groups. **(k)** HOMA-IR of F1 males, n = 8 mice per group. **(l)** Daily food intake of F1 males, n = 6 mice per group. **(m)** Representative pictures of F1 males on HFD. Two-way ANOVA with the Holm-Sidak method was used to analyze time-dependent body weight changes, serum insulin levels, HOMA-β, HOMA-IR, and food intake. Two-sided t-test was used to analyze litter size and birth weight. Data are mean ± S.E.M. * *P* < 0.05 between conF1 and iAsF1 groups under the same conditions.


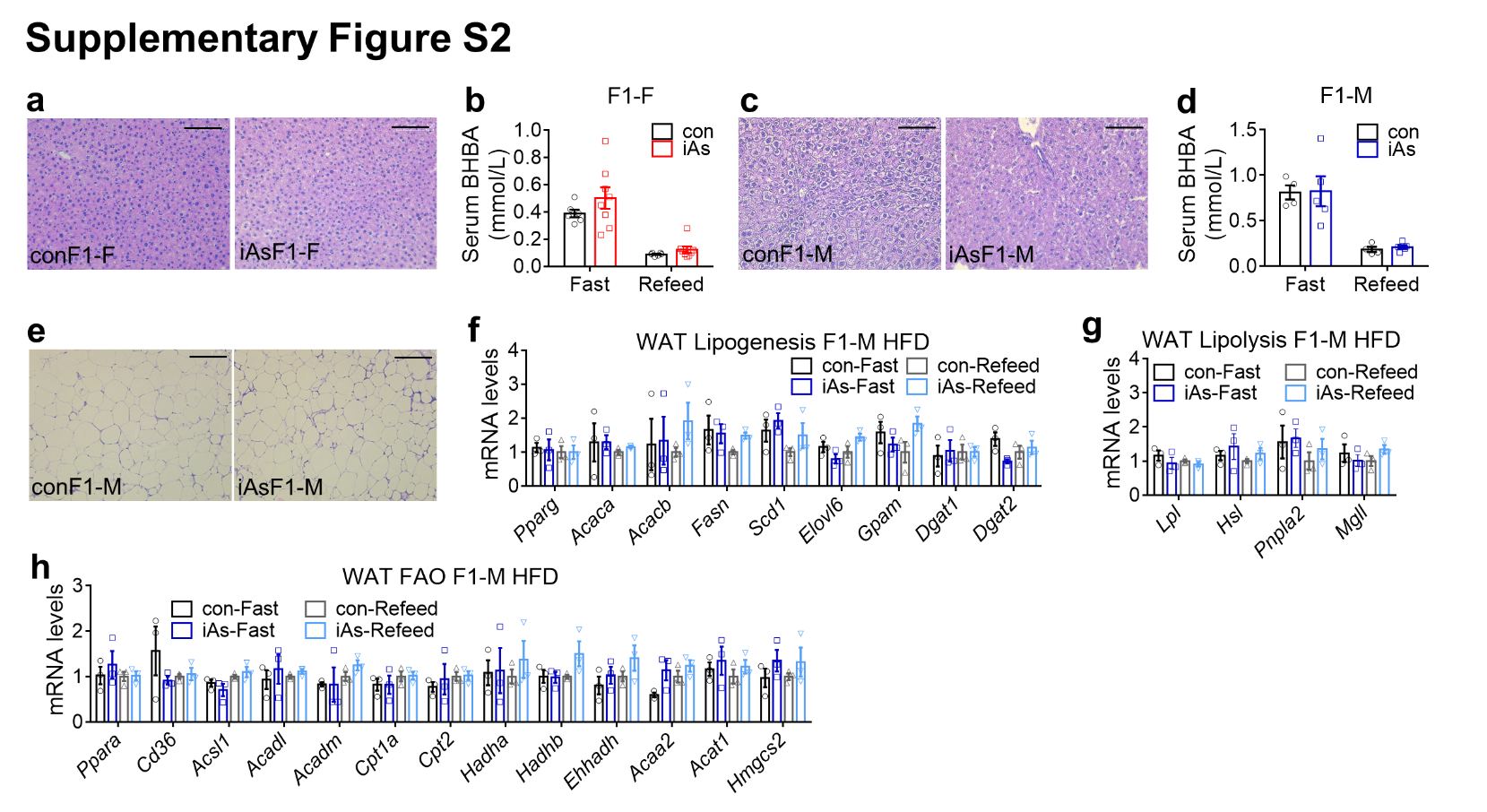


**Supplementary Figure S2. Lipid metabolic phenotype characterizations in F1 offspring.**

**(a)** Hematoxylin and eosin (H&E) staining of livers from F1 females. Scale bar, 100 µm. **(b)** Serum β-hydroxybutyrate (BHBA) levels in F1 females, n = 6 mice for control and n = 8 mice for iAs groups. **(c)** Liver H&E staining from F1 males. Scale bar, 100 µm. **(d)** Serum BHBA levels in F1 males, n = 4 mice for control and n = 5 mice for iAs groups. **(e)** White adipose tissue (WAT) H&E staining from F1 males on HFD. Scale bar, 200 µm. **(f-h)** RT-qPCR analysis of genes in lipogenesis, lipolysis, and fatty acid oxidation (FAO) in the WAT of F1 males on HFD, n = 3 mice. The mean value in the con-Refeed group was set as 1. Two-way ANOVA with the Holm-Sidak method was used to analyze serum BHBA levels and gene expression data. Data are mean ± S.E.M. * *P* < 0.05 between conF1 and iAsF1 groups under the same conditions.


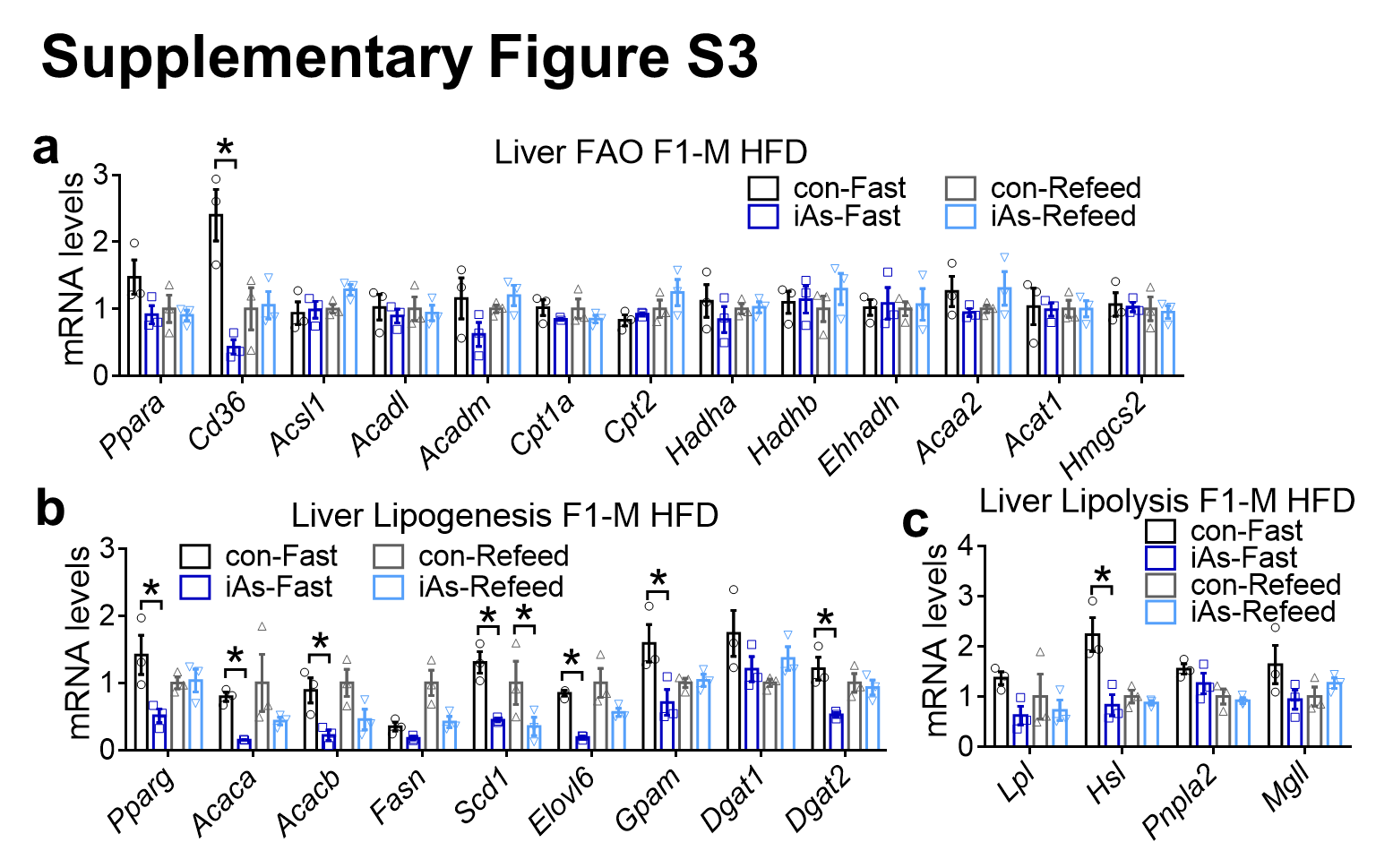


**Supplementary Figure S3. Hepatic gene expression analysis of F1 males on HFD.**

**(a-c)** RT-qPCR analysis of genes in fatty acid oxidation (FAO), lipogenesis, and lipolysis in the liver of F1 males on HFD, n = 3 mice. The mean value in the con-Refeed group was set as 1. Two-way ANOVA with the Holm-Sidak method was used to analyze gene expression data. Data are mean ± S.E.M. * *P* < 0.05 between conF1 and iAsF1 groups under the same conditions.

**
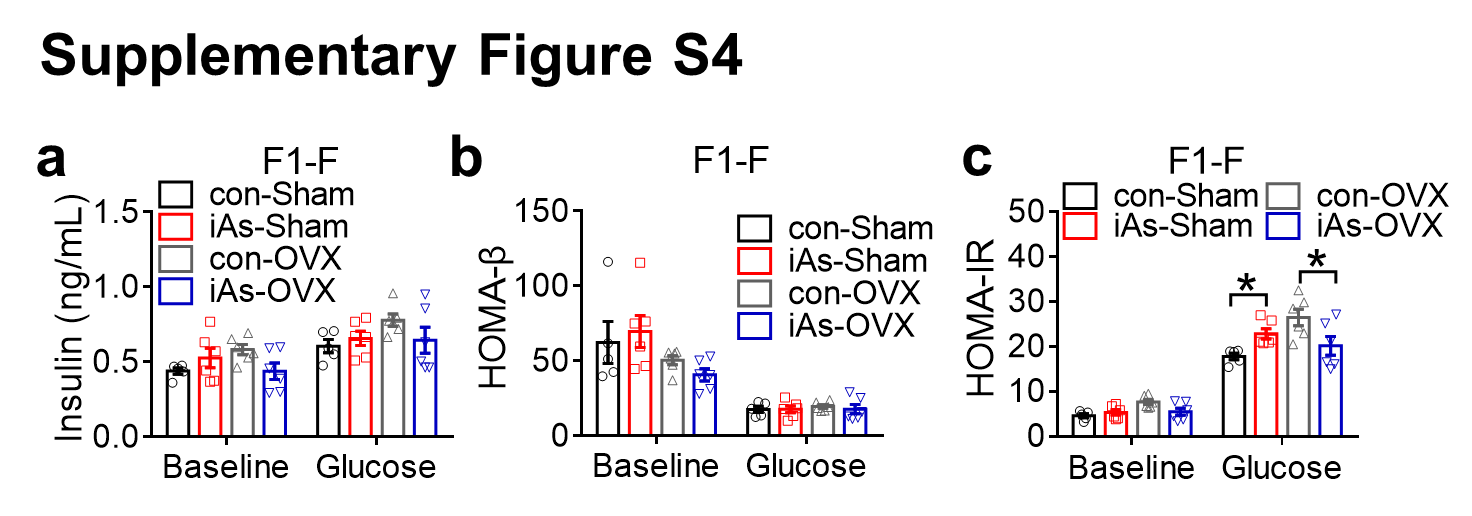
**

**Supplementary Figure S4. Blood parameters of F1 females after sham or OVX.**

**(a-c)** Serum insulin levels, HOMA-β, and HOMA-IR of F1 females after sham or OVX at 23 weeks old, n = 5-6 mice. Two-way ANOVA with the Holm-Sidak method was used to analyze serum insulin levels, HOMA-β, and HOMA-IR. Data are mean ± S.E.M. * *P* < 0.05 between conF1 and iAsF1 groups under the same conditions.


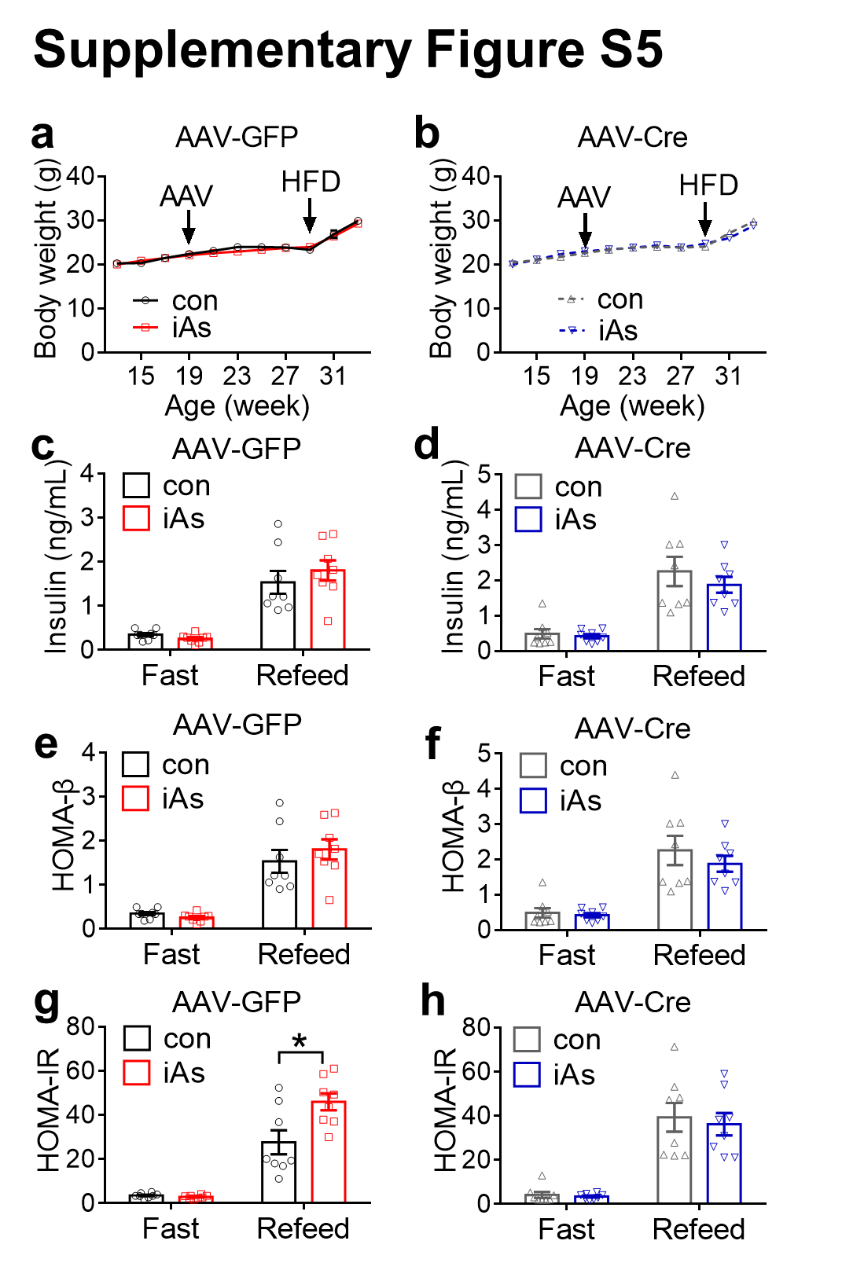


**Supplementary Figure S5. Body weight and blood parameters of F1 females with liver-specific deletion of ERα/β. (a-b)** Body weight of F1 females injected with AAV-GFP or AAV-Cre, n = 8-10 mice. **(c-d)** Serum insulin levels at fasting or refed conditions in F1 females at 28 weeks old injected with AAV-GFP or AAV-Cre, n = 8 mice. **(e-f)** HOMA-β in F1 females injected with AAV-GFP or AAV-Cre, n = 8 mice. **(g-h)** HOMA-IR in F1 females injected with AAV-GFP or AAV-Cre, n = 8 mice. Two-way ANOVA with the Holm-Sidak method was used to analyze time-dependent body weight changes, serum insulin levels, HOMA-β, and HOMA-IR. Data are mean ± S.E.M. * *P* < 0.05 between conF1 and iAsF1 groups under the same conditions.


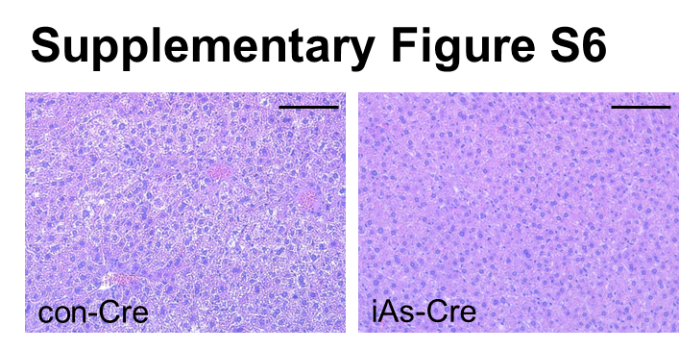


**Supplementary Figure S6. Histology analysis of the liver in F1 females.**

Hematoxylin and eosin (H&E) staining of livers in F1 females injected with AAV-Cre. Scale bar, 100 µm.

**Supplementary Table S1. Primers for RT-qPCR**

| Gene | Forward primer | Reverse primer |
| --- | --- | --- |
| Ppara | AAGCCATCTTCACGATGCTG | TCAGAGGTCCCTGAACAGTG |
| Cd36 | CCTGCAAATGTCAGAGGAAA | GCGACATGATTAATGGCACA |
| Acsl1 | TGCCAGAGCTGATTGACATTC | GGCATACCAGAAGGTGGTGAG |
| Acadl | TCTTTTCCTCGGAGCATGACA | GACCTCTCTACTCACTTCTCCAG |
| Acadm | AGGGTTTAGTTTTGAGTTGACGG | CCCCGCTTTTGTCATATTCCG |
| Cpt1a | CTCCGCCTGAGCCATGAAG | CACCAGTGATGATGCCATTCT |
| Cpt2 | CAGCACAGCATCGTACCCA | TCCCAATGCCGTTCTCAAAAT |
| Hadha | TGCATTTGCCGCAGCTTTAC | GTTGGCCCAGATTTCGTTCA |
| Hadhb | AGCCATGGTGACAGTGTGAG | CAGGAAGTGAAAACAAGCAATG |
| Ehhadh | ATGGCTGAGTATCTGAGGCTG | GGTCCAAACTAGCTTTCTGGAG |
| Acaa2 | CTGCTACGAGGTGTGTTCATC | AGCTCTGCATGACATTGCCC |
| Acat1 | CAGGAAGTAAGATGCCTGGAAC | TTCACCCCCTTGGATGACATT |
| Hmgcs2 | GAAGAGAGCGATGCAGGAAAC | GTCCACATATTGGGCTGGAAA |
| Pparg | GGAAGACCACTCGCATTCCTT | GTAATCAGCAACCATTGGGTCA |
| Acaca | ATGGGCGGAATGGTCTCTTTC | TGGGGACCTTGTCTTCATCAT |
| Acacb | GATGGAGCGCATACACTTGA | CCGAGTTTGTCACTCGGTTT |
| Fasn | TACAGGAGTTCTGGGCCAAC | GACCGCTTGGGTAATCCATA |
| Scd1 | GCTCTACACCTGCCTCTTCG | GCCGTGCCTTGTAAGTTCTG |
| Elovl6 | AATGGATGCAGGAAAACTGG | AACTTGGCTCGCTTGTTCAT |
| Gpam | ACAGTTGGCACAATAGACGTTT | CCTTCCATTTCAGTGTTGCAGA |
| Dgat1 | TCCGTCCAGGGTGGTAGTG | TGAACAAAGAATCTTGCAGACGA |
| Dgat2 | GCGCTACTTCCGAGACTACTT | GGGCCTTATGCCAGGAAACT |
| Lpl | GCCCGAGGTTTCCACAAATA | GCTGAAGTAGGAGTCGCTTATC |
| Hsl | CCAGCCTGAGGGCTTACTG | CTCCATTGACTGTGACATCTCG |
| Pnpla2 | GGATGGCGGCATTTCAGACA | CAAAGGGTTGGGTTGGTTCAG |
| Mgll | CGGACTTCCAAGTTTTTGTCAGA | GCAGCCACTAGGATGGAGATG |
| Pcx | CAGTGGCTGTCTACTCGGAG | CCGCATCTACACCATTTTCCT |
| Pck1 | CCACAGCTGCTGCAGAACA | GAAGGGTCGCATGGCAAA |
| G6pc | CGACTCGCTATCTCCAAGTGA | GTTGAACCAGTCTCCGACCA |
| ERα | CCCGCCTTCTACAGGTCTAAT | CTTTCTCGTTACTGCTGGACAG |
| ERβ | TCTGGGTATCATTACGGTGTCTGGT | TCTCTCCTGGATCCACACTTGACC |
| 18S | AGTCCCTGCCCTTTGTACACA | CGATCCGAGGGCCTCACTA |
